# Supplementary material for: Visualizing acyl carrier protein interactions within a crosslinked type I polyketide synthase
Source: Nat Commun. 2025 Aug 21;16:7798. doi: 10.1038/s41467-025-63024-x (PMC12370991; doi:10.1038/s41467-025-63024-x)
Supplement: Supplementary file 1 — Supplementary Information [file 41467_2025_63024_MOESM1_ESM.pdf]

Supplemental Information

Visualizing Acyl Carrier Protein Interactions within a Crosslinked Type I Polyketide Synthase

Ziran Jiang†, Graham W. Heberlig†, Jeffrey A. Chen, Jennifer Huynh, James J. La Clair, Michael D. Burkart*

Department of Chemistry and Biochemistry, University of California, San Diego, 9500 Gilman Drive, La Jolla CA 92093-0358, United States

* Corresponding author: Michael D. Burkart (UC San Diego) email: mburkart@ucsd.edu

† These authors contributed equally to the work.

Table of Contents

Supplementary Figs. 1-52 S2-S54
Supplementary Table 1 S55
Supplementary Table 2 S56-57

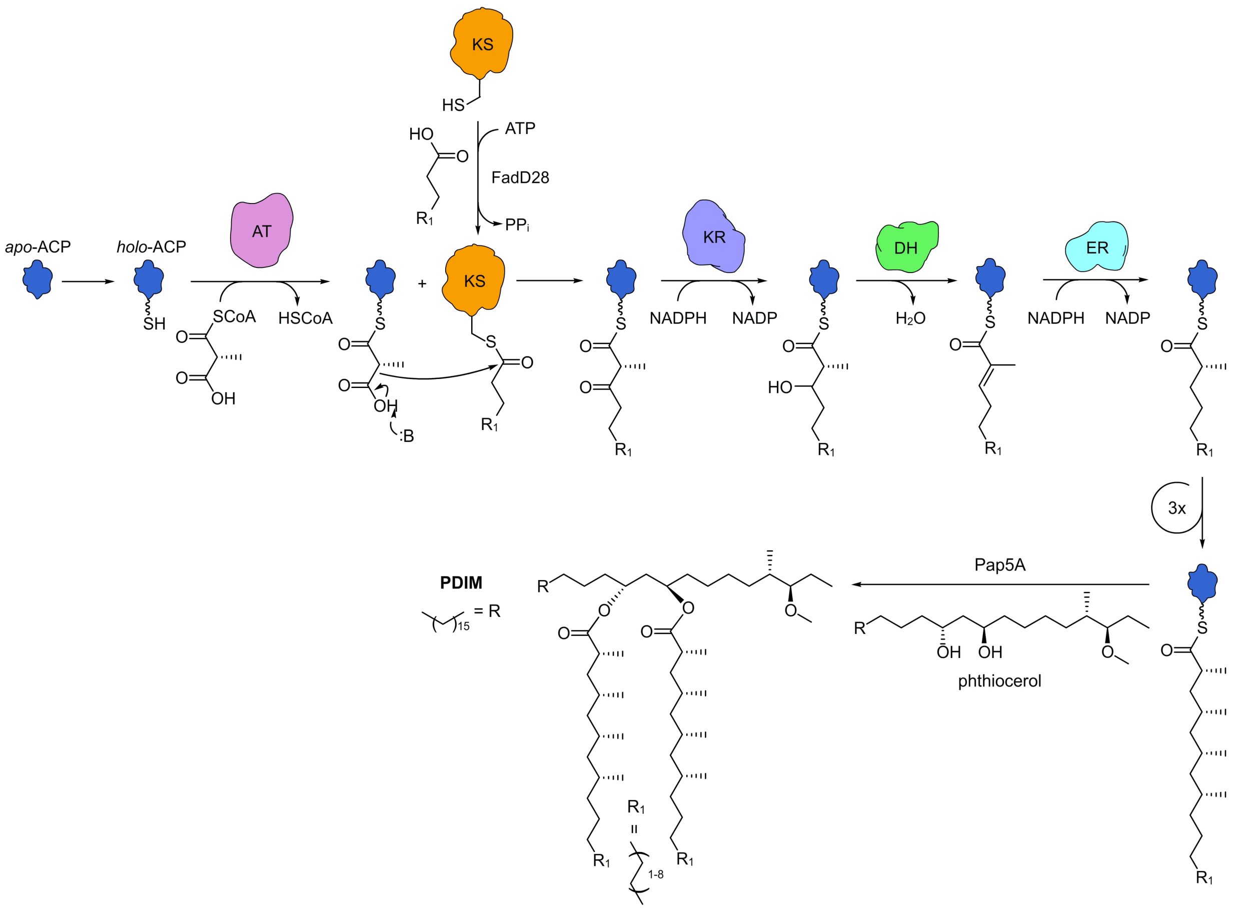

Supplementary Fig. 1 | The biosynthesis of mycocerosic acid and its downstream incorporation in phthiocerol dimycocerosate (PDIM) by FadD28, mycocerosic acid synthase (MAS), and PapA5 from *Mycobacterium tuberculosis*. Straight chain fatty acids (C6-C20) are adenylated by AMP ligase FadD28 and loaded onto ketosynthase (KS) of MAS. The *holo*-acyl carrier protein (ACP) of MAS is loaded with a methylmalonyl extension unit by an acyl transferase (AT) in the transacylation step. The methylmalonyl ACP performs a decarboxylative Claisen-like condensation with the previously loaded acyl chain (C6-C20) in KS in the condensation step. The extended acyl chain is then shuttled on an ACP to ketoreductase (KR) for β-keto reduction using NADPH. The resulting β-hydroxy group is then dehydrated by dehydratase (DH) to give β-enoyl acyl ACP. Sequentially, the β-enoyl group is further reduced by enoylreductase (ER) into a fully saturated branched acyl ACP using NADPH. The fully saturated branched acyl chain is then reloaded onto the KS for 1-4 iterations to produce mycocerosic acids (different chain lengths observed). PapA5 can promote the diesterification between mycocerosates from ACP and phthiocerol to produce PDIM which will be incorporated into the cell envelop of *M. tuberculosis*. Protomers are not used in this figure and hence domains are named accordingly.

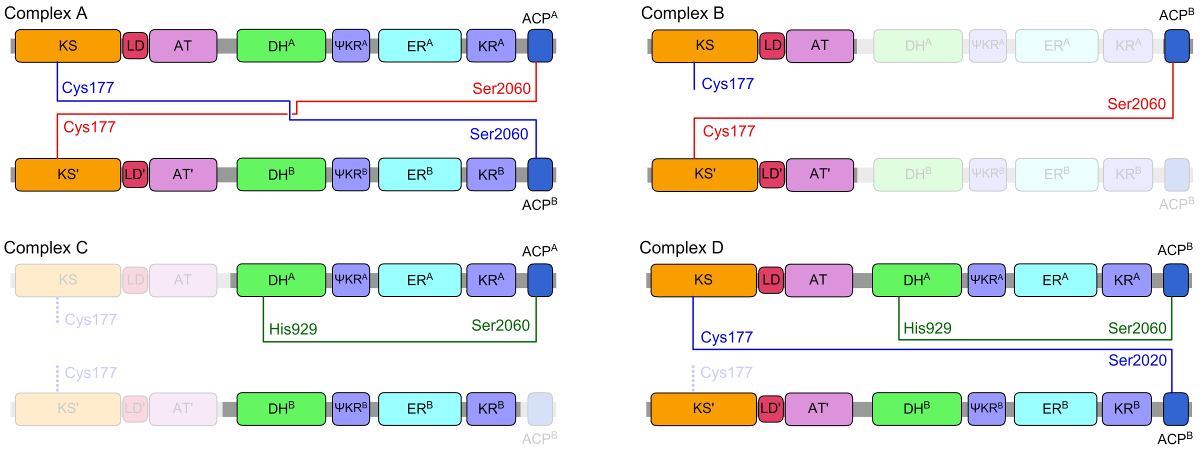

Supplementary Fig. 2 | Intramolecular crosslinking by **1** in MAS. In complex A, ACP crosslinked to KS domains, and all domains are visualized by density map. This demonstration does not exclude crosslinking within the same protomer. In complex A, all domains were visualized. Domains with grey shading are present but disordered. In complex B, ACP crosslinked with KS or KS' while the modifying compartment was not visualized. In complex C, ACP crosslinked at DH, while only DH^A-$ΨKR^A$-ER^A-KR^A-ACP^A and DH^B-$ΨKR^B$-ER^B-KR^B were visualized. In complex D, although molecular structure was not modeled due to low resolution, map density covered all catalytic domains including both ACPs which crosslinked with DH and KS. While one ACP is crosslinked at DH in one protomer, the other ACP is allowed to crosslink with KS from the same or the other protomer.

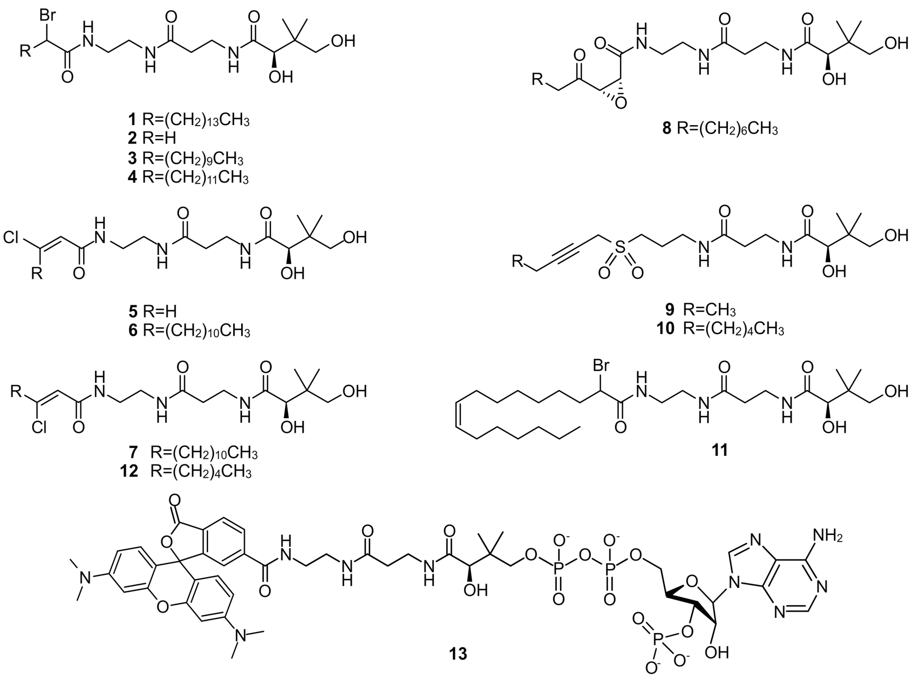

Supplementary Fig. 3 | Structures of crosslinkers **1-12** and fluorescent CoA analogue **13**. The syntheses of these crosslinkers are described in previous published methods.[20,32,56] In general, crosslinkers **1-4**, and **11** containing the α-bromine motif warhead react with the reactive residue cysteine in ketosynthases as well as the reactive residue histidine in dehydratase. Crosslinkers **5**, **6**, **7**, **8**, and **12** containing the chloroacrylate or reactive epoxide motif also target the reactive residue cysteine in ketosynthases. Crosslinkers **9** and **10** containing the sulfone motif react with the reactive histidine of dehydratase. These crosslinking pantetheine analogs can be converted into CoA analogs (Supplementary Fig. 4B) by CoaA, CoaD, and CoaE. The loading of CoA analogs (primed crosslinkers) onto ACP reactive residue serine is accomplished by Sfp.[25]

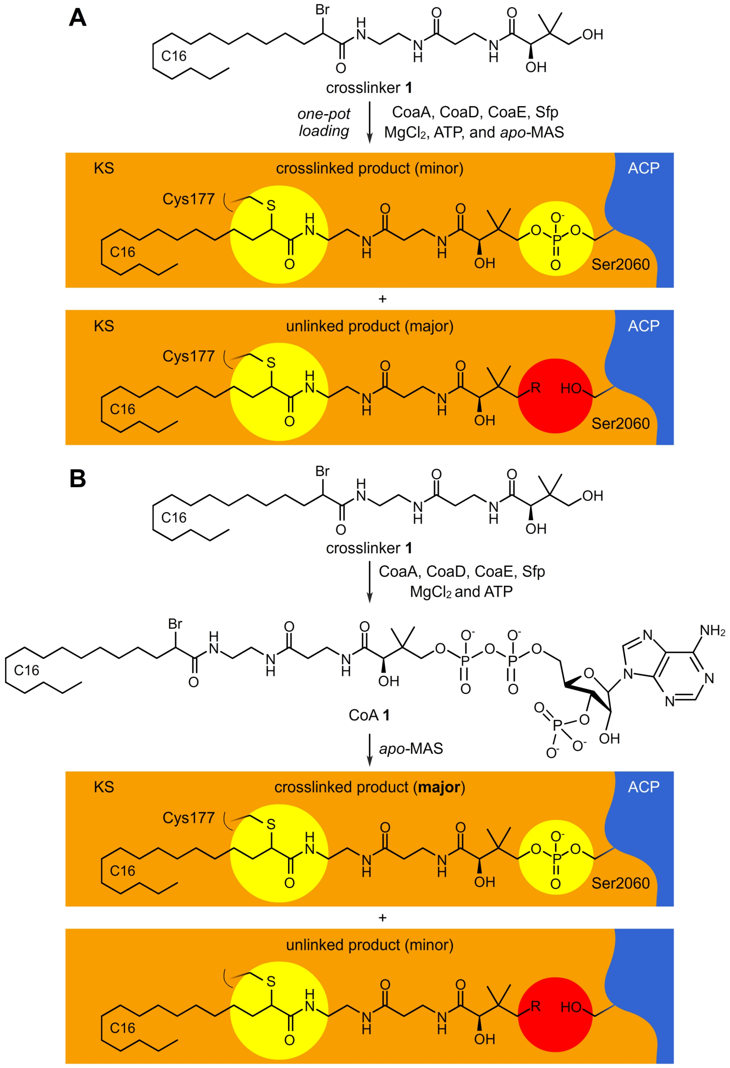

Supplementary Fig. 4 | Optimization of *in cis* MAS crosslinking. (**A**) Crosslinking *in cis* in MAS will produce uncrosslinkable shunt products which will reduce the yield. "R" is a phospho or CoA group. (**B**) Crosslinking *in cis* in MAS with optimized one pot reaction. Crosslinker **1** is primed with CoaA, CoaD, CoaE, ATP, Sfp, and $MgCl_2$. Then the primed **1** will be added into MAS to reach optimal yield. "R" is a phospho or CoA group. The detailed crosslinker loading and crosslinking procedures are described in the general experiment methods section.

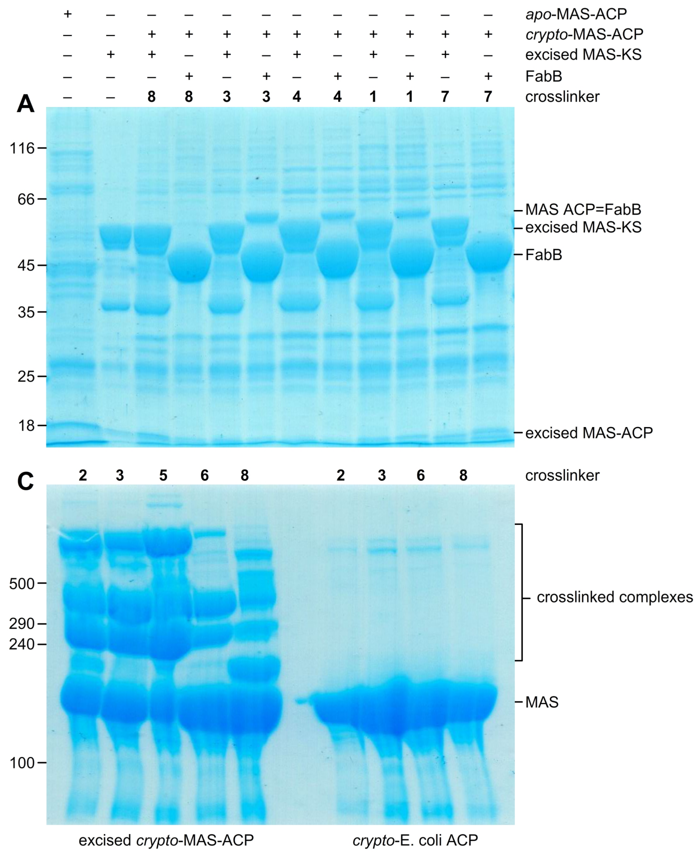

Supplementary Fig. 5 | Exploring crosslinking *in trans*. (**A**) 12% SDS-PAGE analysis of *in trans* crosslinking between excised ACP and excised KS or FabB by crosslinkers **1**, **3**, **4**, **7**, **8**. Mild crosslinking was observed between excised ACP and FabB by **1**, **3**, and **4** by an upshifted band while no crosslinking was observed between excised ACP and excised KS. (**B**) 12% SDS-PAGE analysis of *in trans* crosslinking of excised ACP loaded with **2**, **3**, **5**, **6**, or **8** with excised KS-LD-AT. Only moderate crosslinking was observed with **2**. (**C**) SDS-PAGE (6%) analysis of *in trans* crosslinking between full MAS (including ACP) and excised ACP (loaded with **2**, **3**, **5**, **6**, or **8**) or *E. coli* AcpP (loaded with **2**, **3**, **6**, or **8**). In contrast to crosslinking with (**A**) excised KS or (**B**) excised KS-LD-AT, excised ACP was able to crosslink with full MAS by **2**, **3**, **5**, **6**, or **8** indicated by upshifted bands. Although buffer exchange was performed to remove most of the free crosslinkers (not loaded onto ACP), the residual crosslinkers along with excised ACP loaded with crosslinkers were able to give intramolecular crosslinking and intermolecular crosslinking. Therefore, a series of upshifted bands were observed for both intramolecularly and intermolecularly crosslinked complexes. Detailed SDS-PAGE analysis regarding to the crosslinked complexes can be found in Supplementary Figs. 7 and 45. On the other hand, no crosslinking was observed between *E. coli* AcpP and full MAS with **2**, **3**, **6**, or **8**.

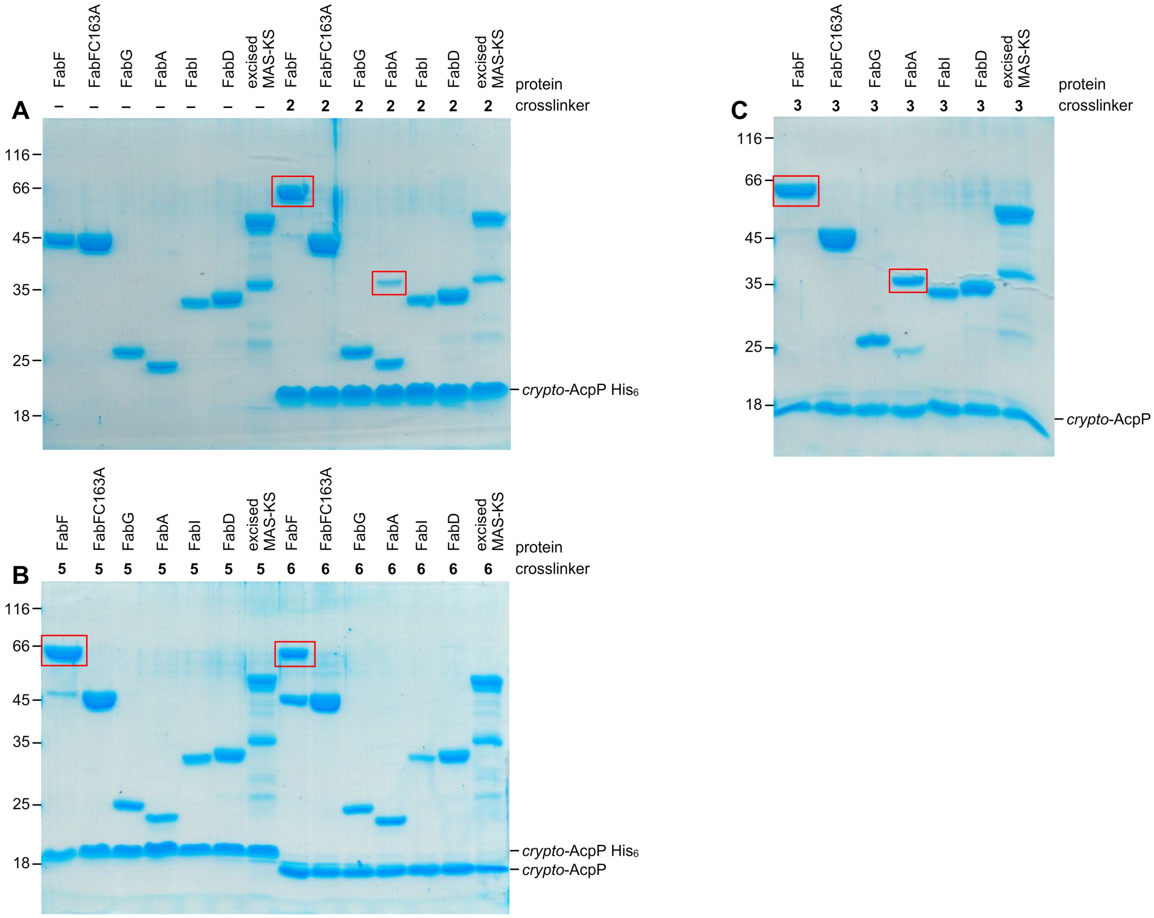

Supplementary Fig. 6 | Specificity study of crosslinker 2, 3, 5, and 6 in *E. coli* Type II FAS system. This provides an *in trans* crosslinking experiment model for testing the specificity of crosslinkers since *in cis* crosslinking experiment in Type I system for crosslinker specificity presents challenges. *E. coli* AcpP (C-terminal His6-tag) loaded with (**A**) **2** or (**B**) **5** and AcpP (native) loaded with (**B**) **6** or (**C**) **3** were tested with a panel of partner proteins including FabF (KS), FabFC163A (active site residue mutant), FabG (KR), FabA (DH), FabI (ER), FabD (AT), and excised KS. Upshifted bands with higher molecular weight (highlighted in red) are the corresponding crosslinked complex between AcpP and partner proteins. While all crosslinkers tested showed crosslinking with FabF, only **2** and **3** carrying a highly reactive α-bromoamide crosslinked with FabA.

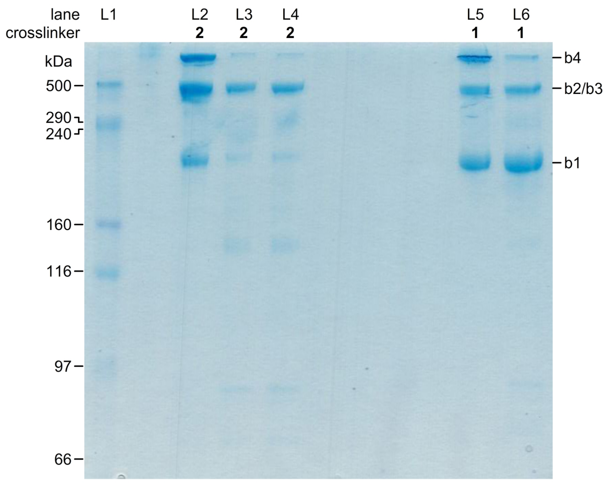

Supplementary Fig. 7 | SDS-PAGE (6%) demonstrates the MAS crosslinking *in cis* using crosslinkers **1** and **2**. Lanes 2-4 and 5-6 are consecutive fractions from FPLC purification. Lane 5 was selected for single particle analysis. The purified crosslinked complexes were identified as four bands, b1, b2, b3, and b4. Complex C, uncrosslinked MAS monomer, and intra-protomerically crosslinked MAS monomer were assigned to b1. Complex B was assigned to b2, and complexes A and D were assigned to b3. Intermolecular crosslinking of MAS (oligomer after denaturing) was observed as b4. Crosslinking yield was calculated by the band intensity of b1-b3 over the band intensity of b1-b4. A throughout analysis of b1, b2, b3, and b4 can be found in Supplementary Fig. 45.

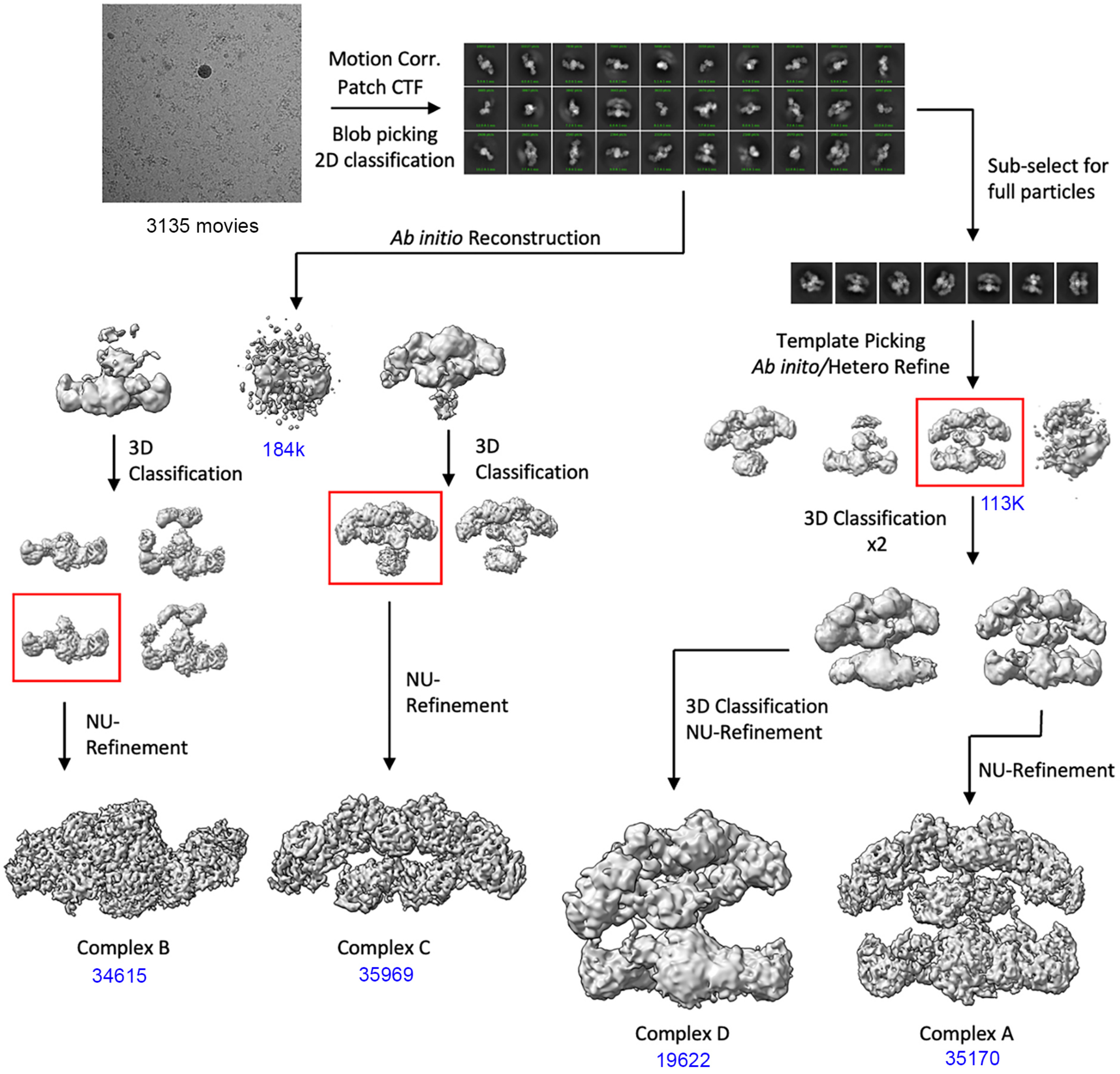

Supplementary Fig. 8 | Data processing workflow showing an overview of the steps taken to generate each of the four cryo-EM maps used in this study. Particle counts provided in blue.

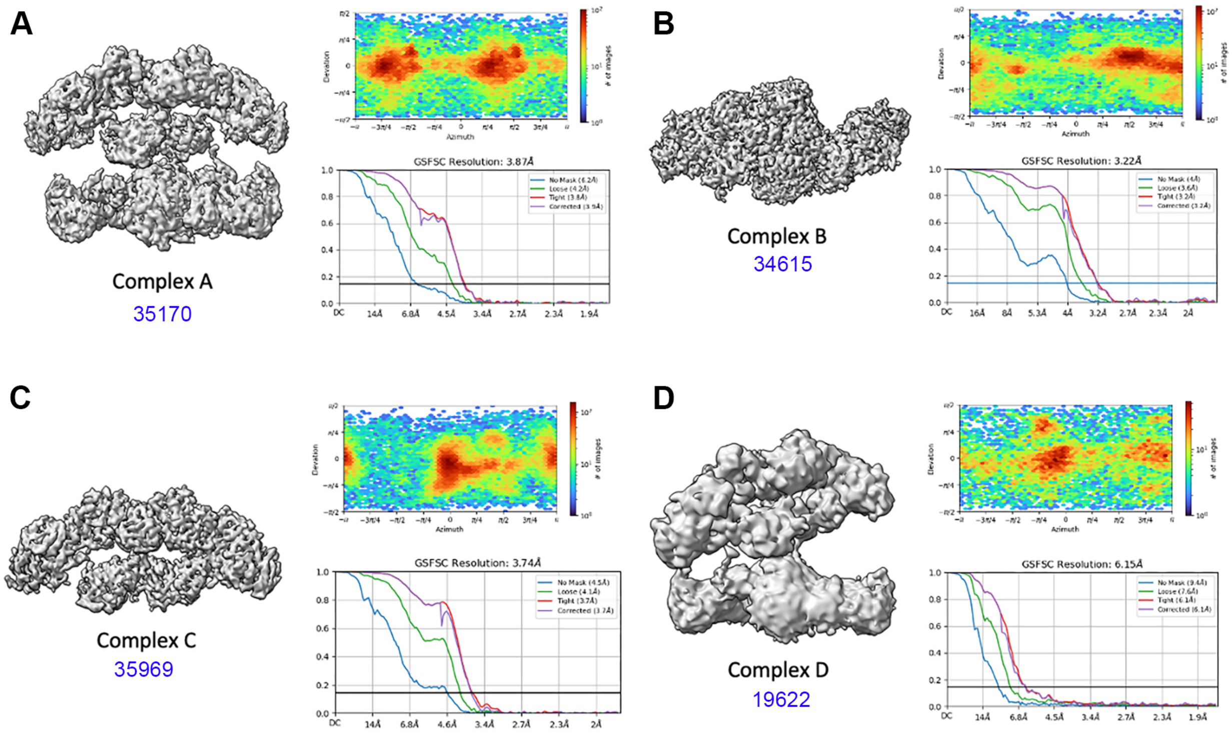

Supplementary Fig. 9 | Structural summary with viewing direction distribution and gold standard FSC curves. (**A**) Summary for complex A. (**B**) Summary of complex B. (**C**) Summary of complex C. (**D**) Summary of complex D. Particle counts provided in blue.

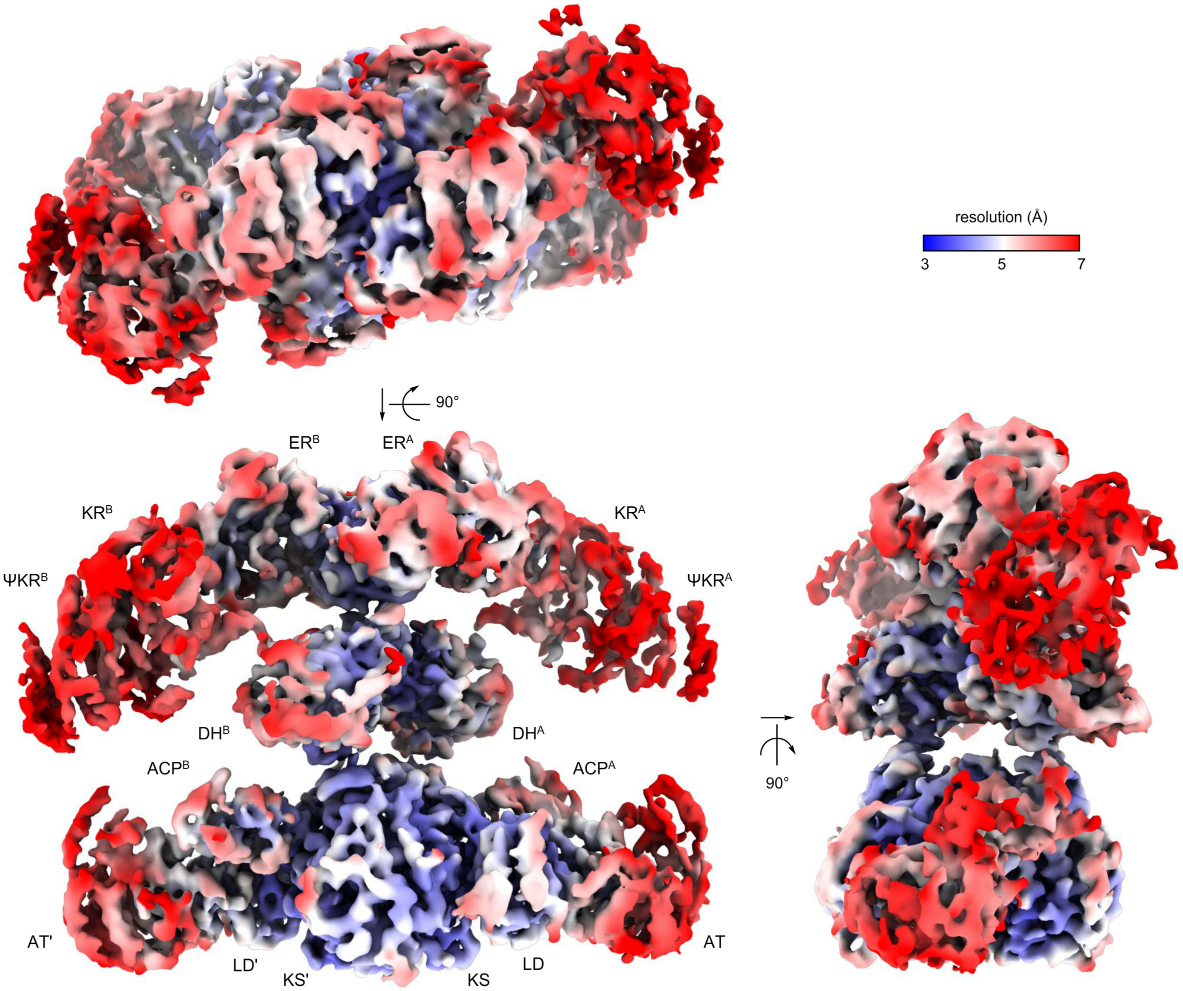

Supplementary Fig. 10 | Cryo-EM map of MAS complex A (contour level = 0.088). This figure depicts the front, top, and side views of the density map of MAS complex A colored based on the local resolution. Regions with ACP bound and crosslinked demonstrated higher resolution. Due to the structural dynamics of MAS, the edge of the non-crosslinked domains was observed in reduced resolution.

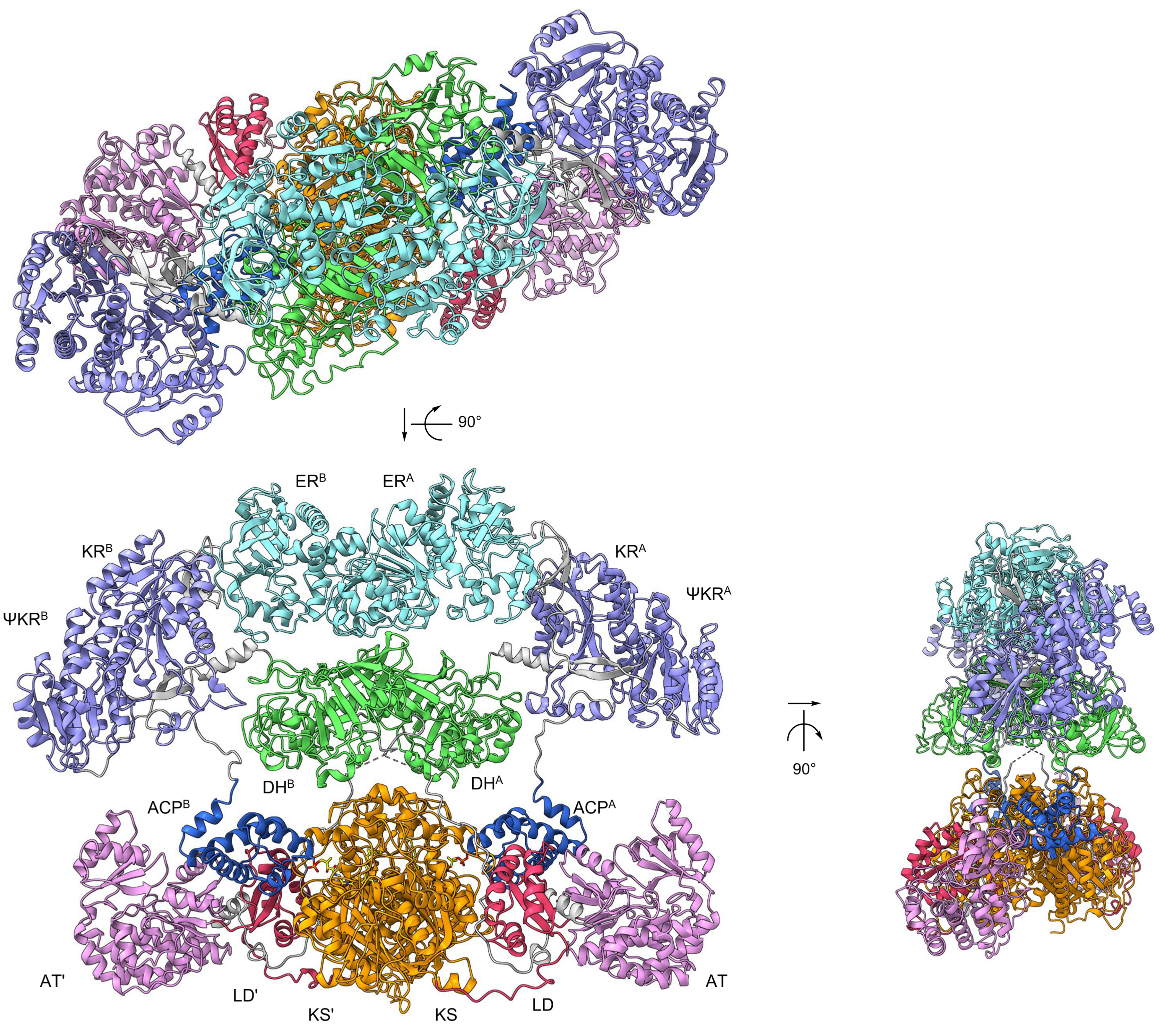

Supplementary Fig. 11 | The architecture of MAS complex A with both ACPs crosslinked at KS domains, respectively. This figure depicts the front, top and side views of MAS complex A with KS in orange, LD in red, AT in pink, DH in green, KR (and ΨKR) in purple, ER in light blue, ACP in blue and inter-domain linkers in grey.

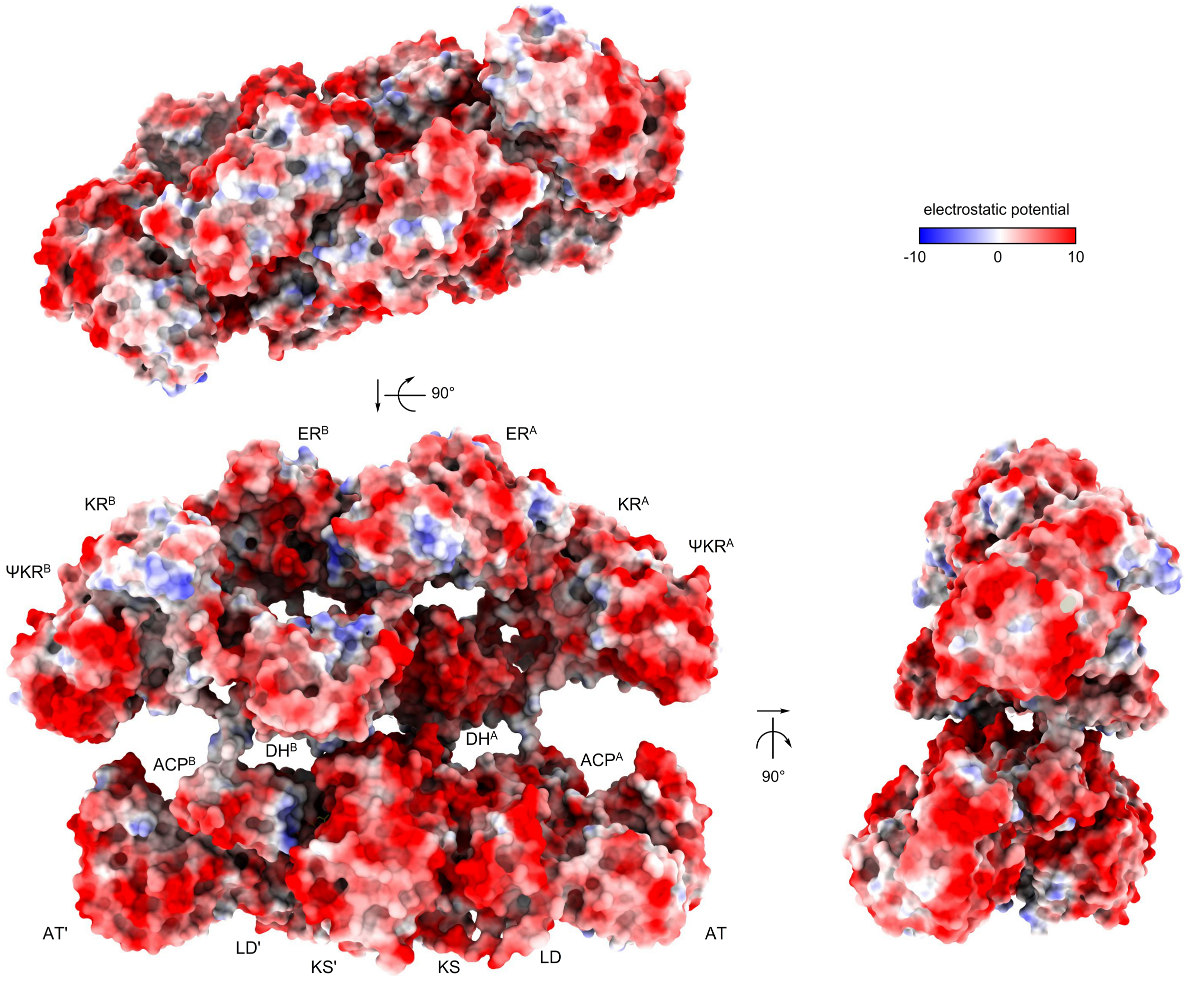

Supplementary Fig. 12 | The architecture of MAS complex A depicting the electrostatic potential of MAS.

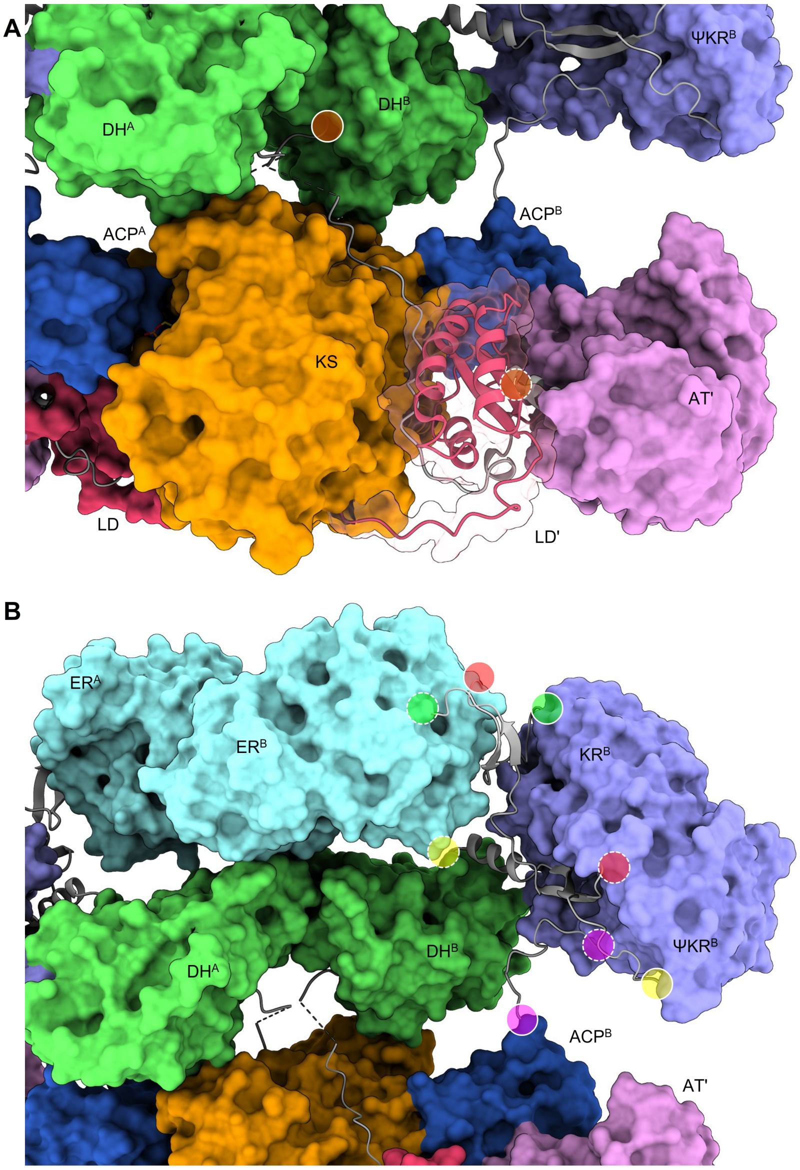

Supplementary Fig. 13 | The linker network of MAS observed in complex A. (**A**) L1 starting from AT (orange dot with dashed line) to DH (orange dot with solid line) wraps around LD (transparent red). (**B**). L2 starts from DH (yellow dot with dashed line) to ΨKR (yellow dot with solid line). L3 starts from ΨKR (red dot with dashed line) to ER (red dot with solid line). L4 starts from ER (green dot with dashed line) to KR (green dot with solid line). L5 starts from KR (purple dot with dashed line) to ACP (purple dot with solid line).

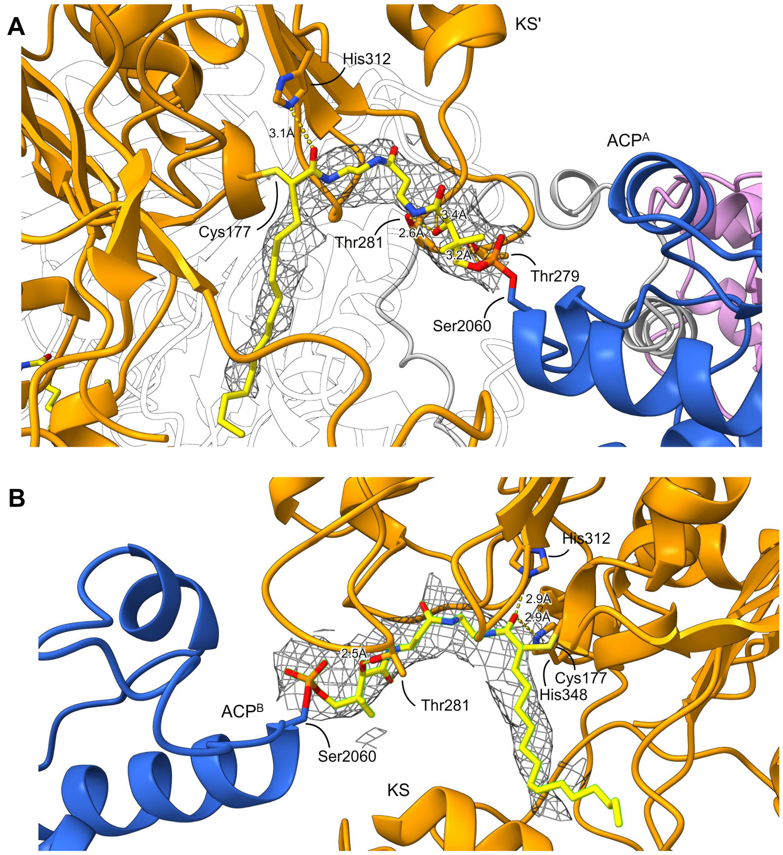

Supplementary Fig. 14 | ACP=KS crosslinks in each protomer in Complex A. (A) In protomer 1 of complex A, crosslinker **1** (yellow) covalently linking Ser2060 of ACP^A to Cys177 of KS', as supported by continuous map density (contour level = 0.088). Crosslinker **1** interacts with Thr279, Thr281, and His348 of KS. **(B)** In protomer 2 of complex A, crosslinker **1** (yellow) covalently linking at Ser2060 of ACP^B to Cys177 of KS supported by continuous map density (contour level = 0.088). Crosslinker **1** interacts with KS in a similar way as that in protomer 1.

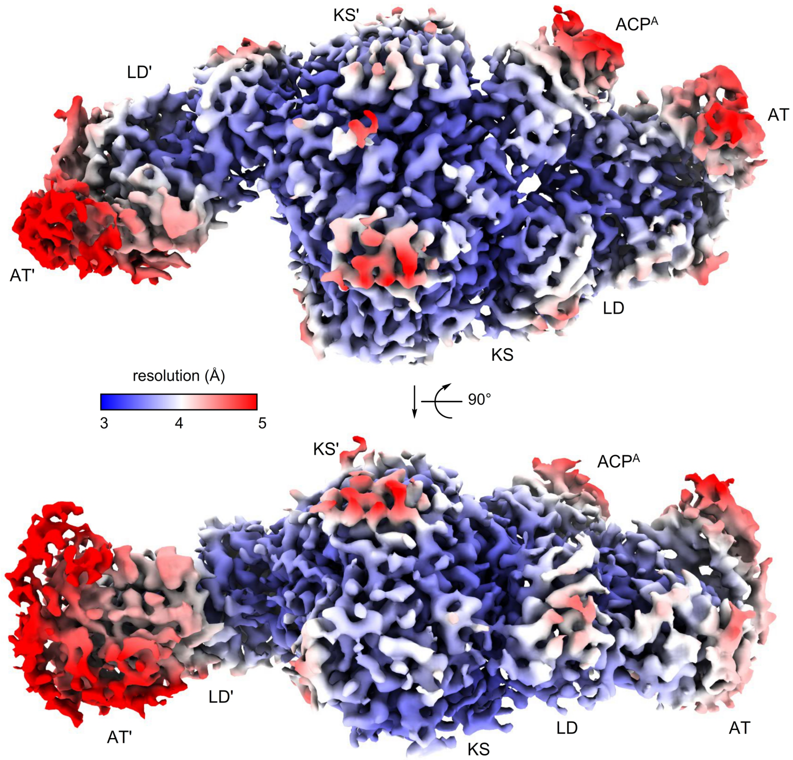

Supplementary Fig. 15 | Density map of complex B. Monomer with ACP^A crosslinked by **1** shows a higher resolution compared with the other monomer that is not crosslinked but only inhibited by **1**. Map displayed with contour level 0.12.

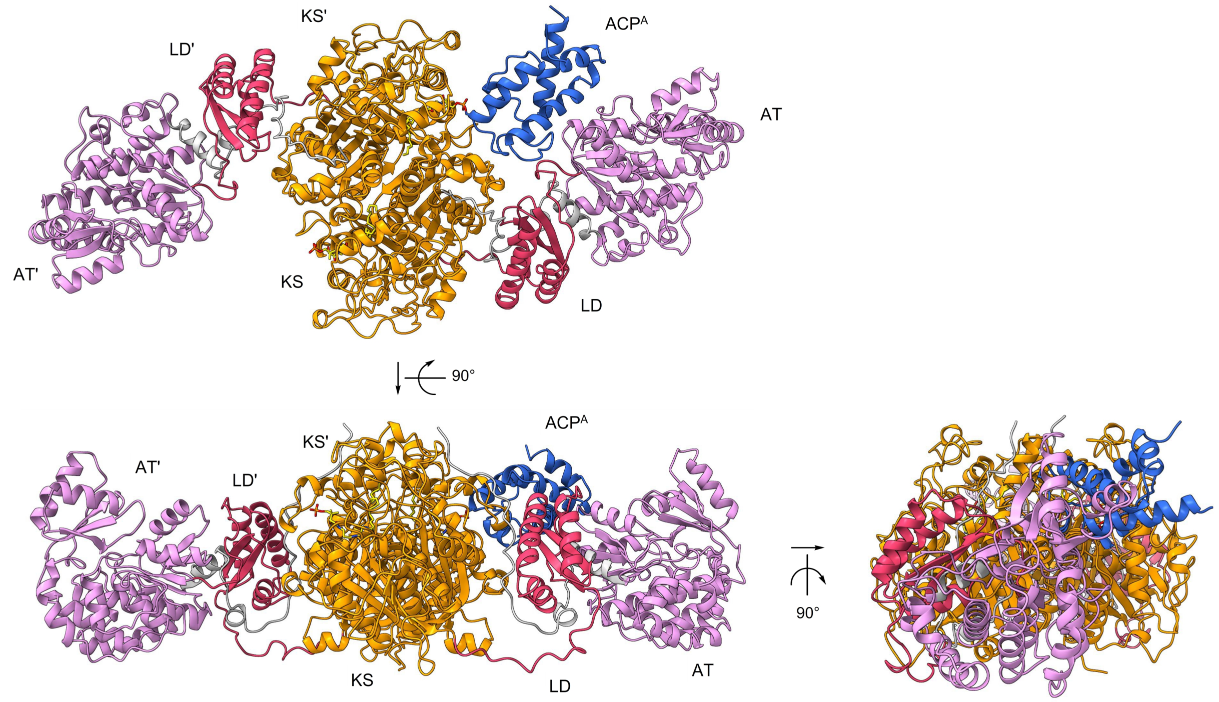

Supplementary Fig. 16 | Complex B. Structure of complex B where ACP crosslinked with KS through crosslinker **1**.

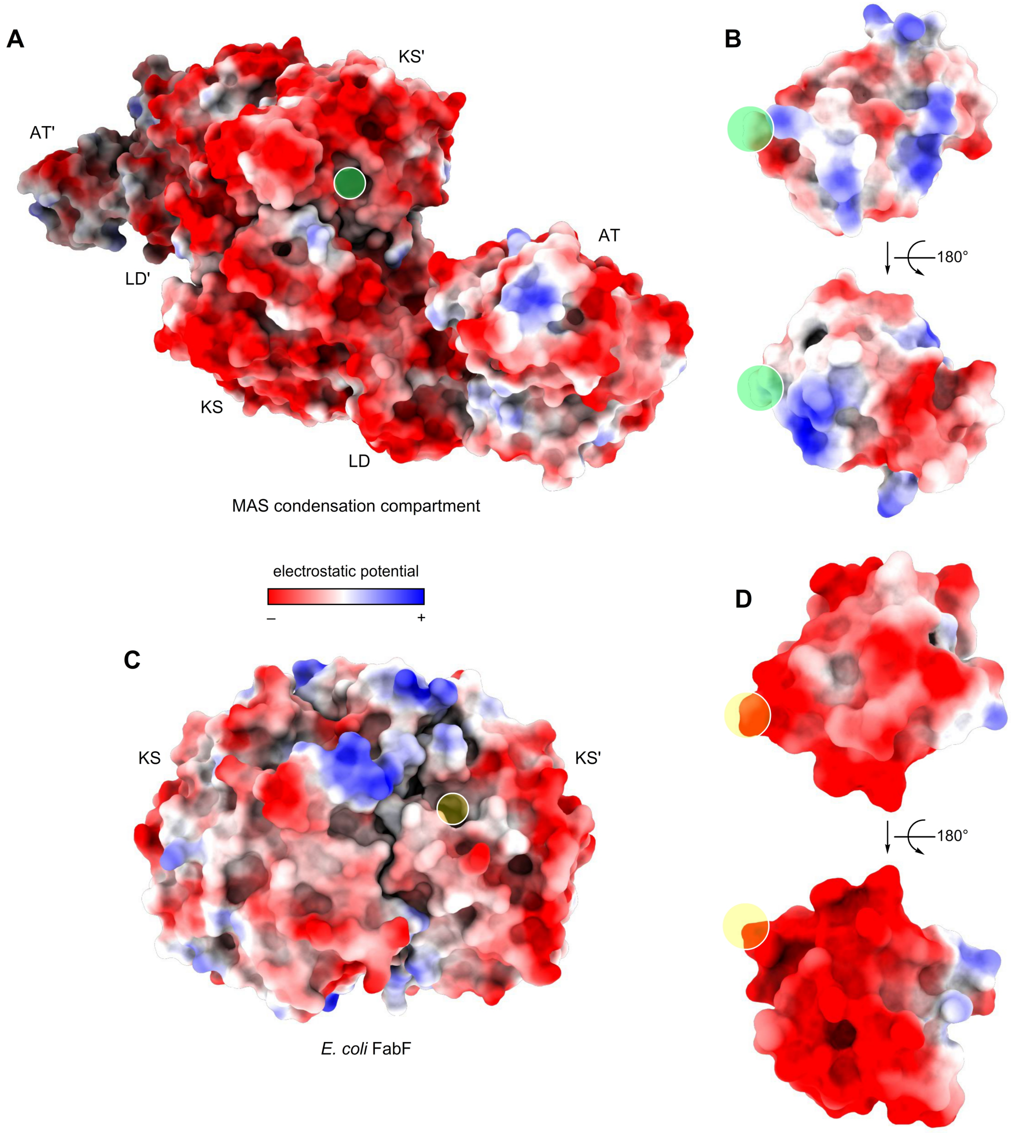

Supplementary Fig. 17 | Electrostatic profile of MAS condensation compartment and FabF. Electrostatic potential profile comparison amongst (**A**) MAS condensation compartment from complex B and (**B**) ACP demonstrates evenly distributed partial charges. Green dot in (**A**) indicates the binding tunnel entrance of KS. And green dots in (**B**) indicate the active site residue of ACP Ser2060. In contrast to MAS, (**C**) *E. coli* FabF (Type II KS) (PDBID: 7L4L) reveals a highly positively charged binding interface, which is opposite to the highly negatively charged *E. coli* AcpP binding interface (PDBID: 7L4L). Yellow dot in (**C**) indicates the binding tunnel entrance of FabF while yellow dots in (**D**) indicate the active site residue Ser36.

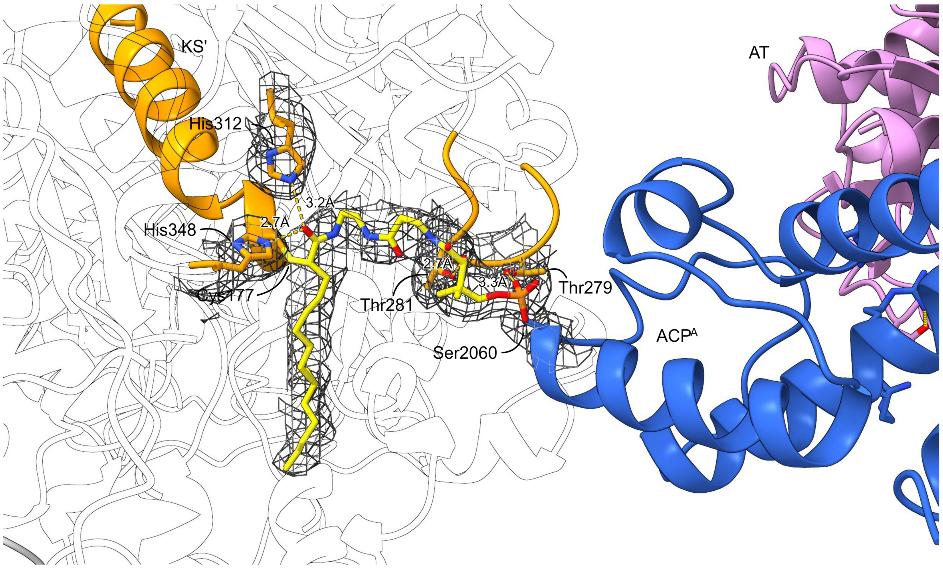

Supplementary Fig. 18 | Active site of KS in complex B occupied with crosslinker 1 tethering ACP. In complex B, **1** (yellow) (contour level of 0.12, map radius of 2.0 Å) forms covalent linkages at KS (orange) active site Cys177 and at ACP^{A} (blue) active site Ser 2060. Crosslinker **1** interacts with His348 and His312, Thr281, and Thr279 of KS through hydrogen bonds.

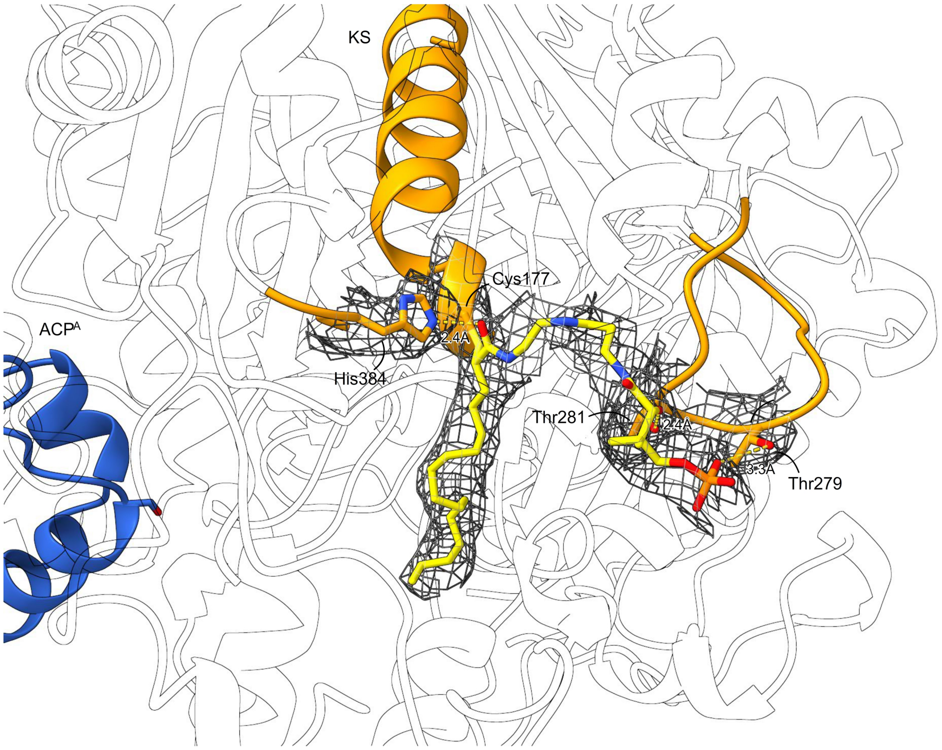

Supplementary Fig. 19 | Active site of KS in complex B inhibited by crosslinker 1. In complex B, unlike the KS that was crosslinked with ACP, the uncrosslinked KS was covalently modified by **1** without ACP tethered (shunt). The occupancy of **1** is supported by the continuous map density (contour level of 0.12, map radius of 2.0 Å). Such observation was due to the highly reactive warhead of **1**.

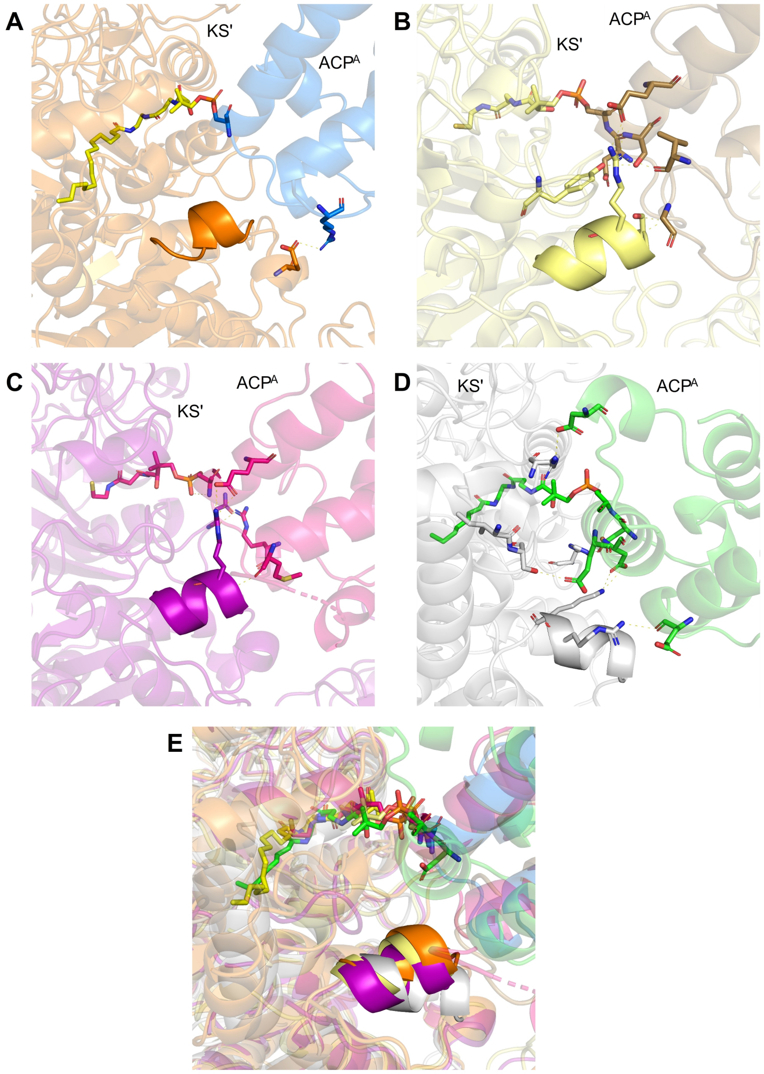

Supplementary Fig. 20 | Structural elucidation of ACP interacting with KS in the presence of substrates in different PKS or FAS systems. (A) MAS ACP^A (blue) loaded with **1** (bright yellow) interacting with KS (orange) through a hydrogen bond between Arg2045 and Glu79. **(B)** DEBS-M1 ACP (PDBID: 7M7F) (dark gold) loaded with a phosphopantetheine bound to DEBS KS (gold) through extensive interactions with ACP helices 1 and 2.[13] **(C)** LSD14 ACP^A (pink) (PDBID: 7S6C) loaded with phosphopantetheine interacts with LSD14 KS (purple) involving helices 1 and 2.[14] **(D)** *E. coli* Type II AcpP (green) (PDBID: 7L4L) loaded with **12** (green) forms interactions with *E. coli* Type II KS FabF (grey) involving helixes 1, 2, and 3.[34] **(E)** Alignment of the structures (excluding ACP^A) to the KS dimeric core of complex B (RMSD= 1.841) reveals that the substrates share the same binding tunnel within KSs. Compared with the KS-ACP interactions in Type I system, *E. coli* Type II system depicts a unique positioning of 6 aa long ACP binding helix to provide more interactions with helixes 1 and 2. Although they share the same canonical KS binding tunnel, the overlay revealed that the Type I ACPs dock onto KSs at a different angle compared with *E. coli* type II AcpP.

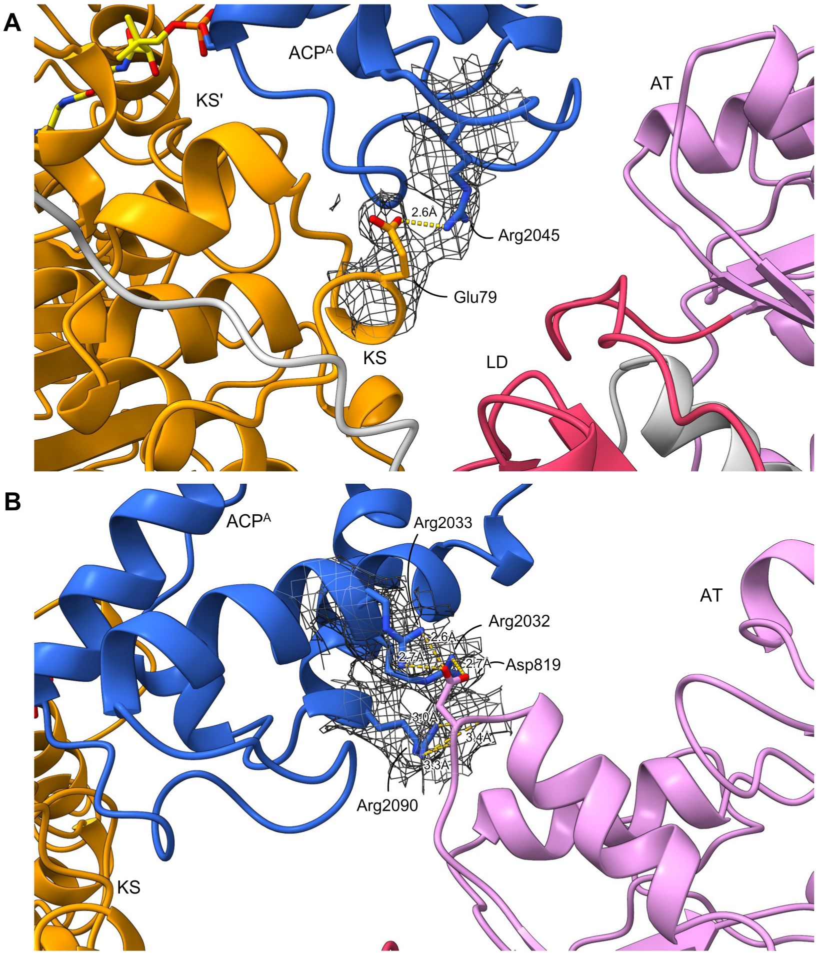

Supplementary Fig. 21 | ACP interactions in complex B. Upon crosslinking at KS, the ACP interactions with KS and AT from the other protomer are supported by map density in complex B (contour level of 0.12, map radius of 2.0 Å).

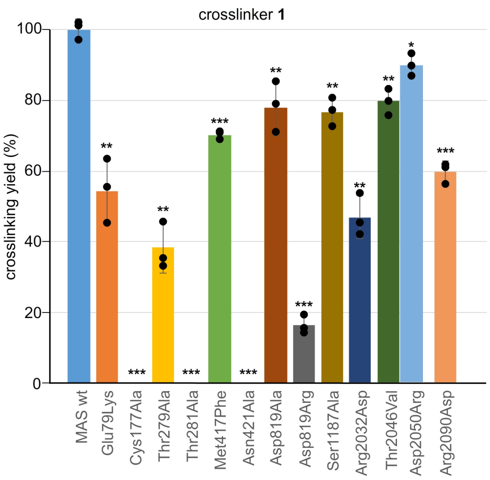

Supplementary Fig. 22 | Crosslinking activity assay. Crosslinking activity assay with **1** was performed on 13 MAS mutants. The yield was determined by the crosslinking with wild type MAS. Crosslinking yields for MAS mutants are relative to the wild type MAS. Bar charts and error bars were generated from the SDS-PAGE gels from Supplementary Figs. 23-27 and quantified with ImageJ.

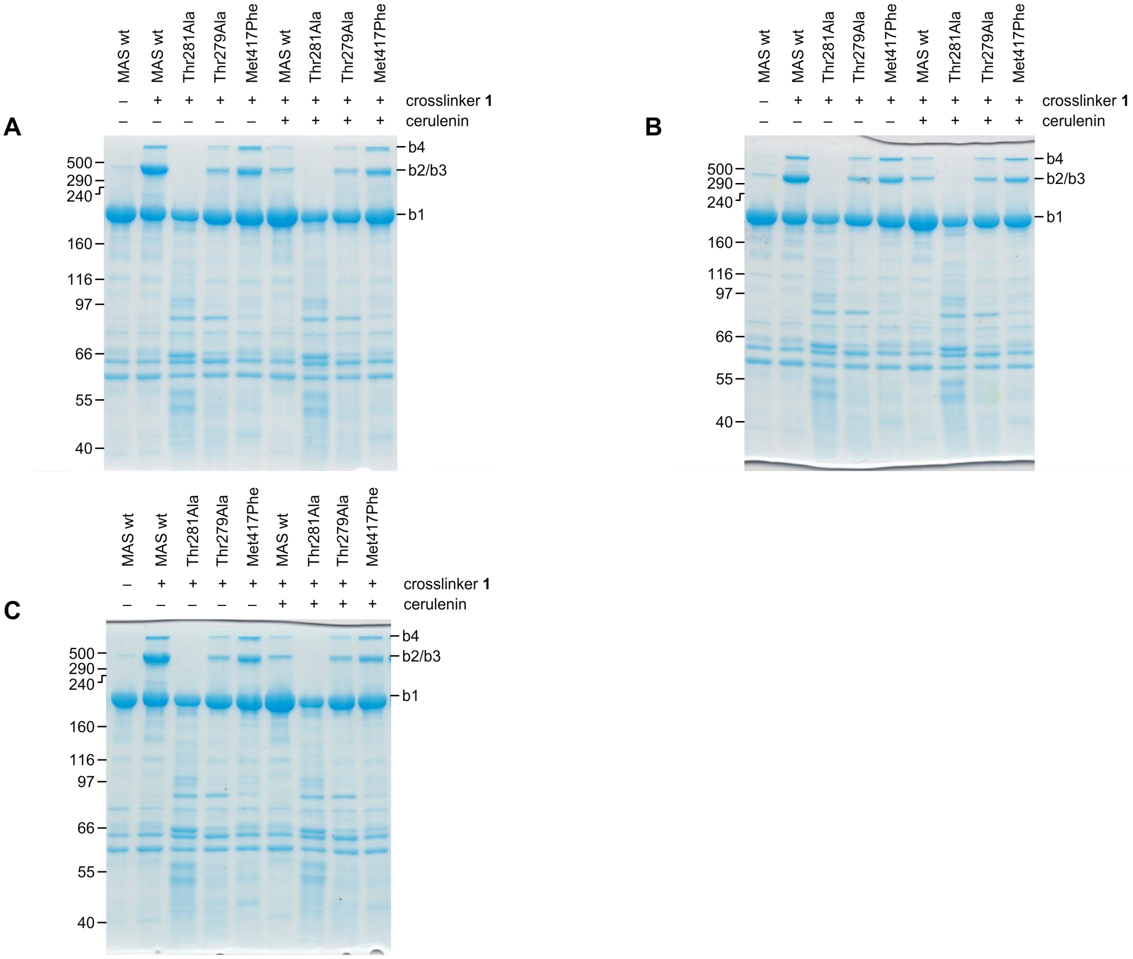

Supplementary Fig. 23 | Crosslinking activity assay SDS-PAGE gels. (**A-C**) Crosslinking activity assay with **1** was performed on MAS wt and MS mutants (Thr281Ala, Thr279Ala, and Met417Phe). These 6% SDS PAGE gels were used to prepare the data in Supplementary Fig. 22. The crosslinked complexes were identified as four bands, b1, b2, b3, and b4. Complex C, uncrosslinked MAS monomer, and intra-protomerically crosslinked MAS monomer were assigned to b1. Complex B was assigned to b2, and complexes A and D were assigned to b3. Intermolecular crosslinking of MAS (oligomer after denaturing) was observed as b4. A throughout analysis of b1, b2, b3, and b4 can be found in Supplementary Fig. 45.

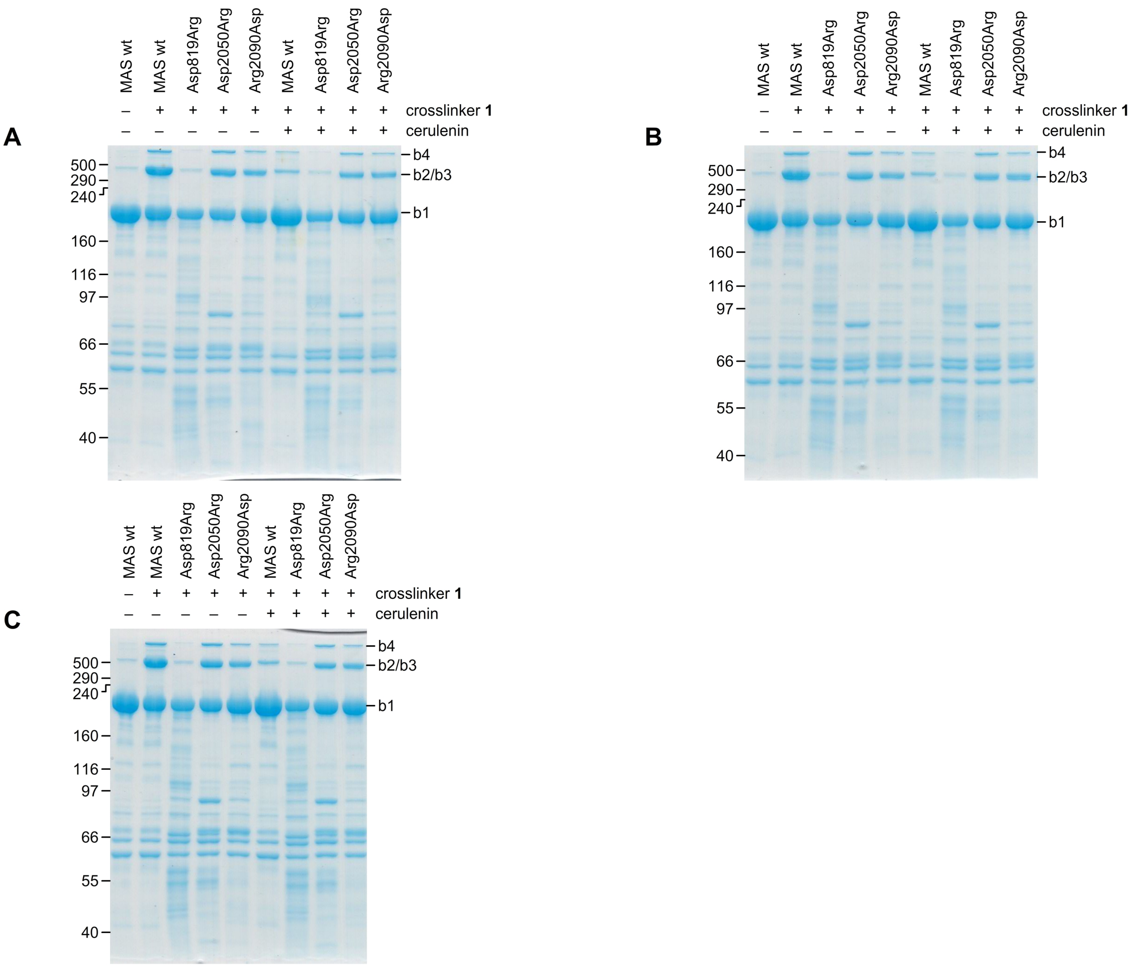

Supplementary Fig. 24 | Crosslinking activity assay SDS-PAGE gels. (**A-C**) Crosslinking activity assay with **1** was performed on MAS wt and MS mutants (Asp819Arg, Asp2050Arg and Arg2090Asp). These 6% SDS PAGE gels were used to prepare the data in Supplementary Fig. 22. The crosslinked complexes were identified as four bands, b1, b2, b3, and b4. Complex C, uncrosslinked MAS monomer, and intra-protomerically crosslinked MAS monomer were assigned to b1. Complex B was assigned to b2, and complexes A and D were assigned to b3. Intermolecular crosslinking of MAS (oligomer after denaturing) was observed as b4. A throughout analysis of b1, b2, b3, and b4 can be found in Supplementary Fig. 45.

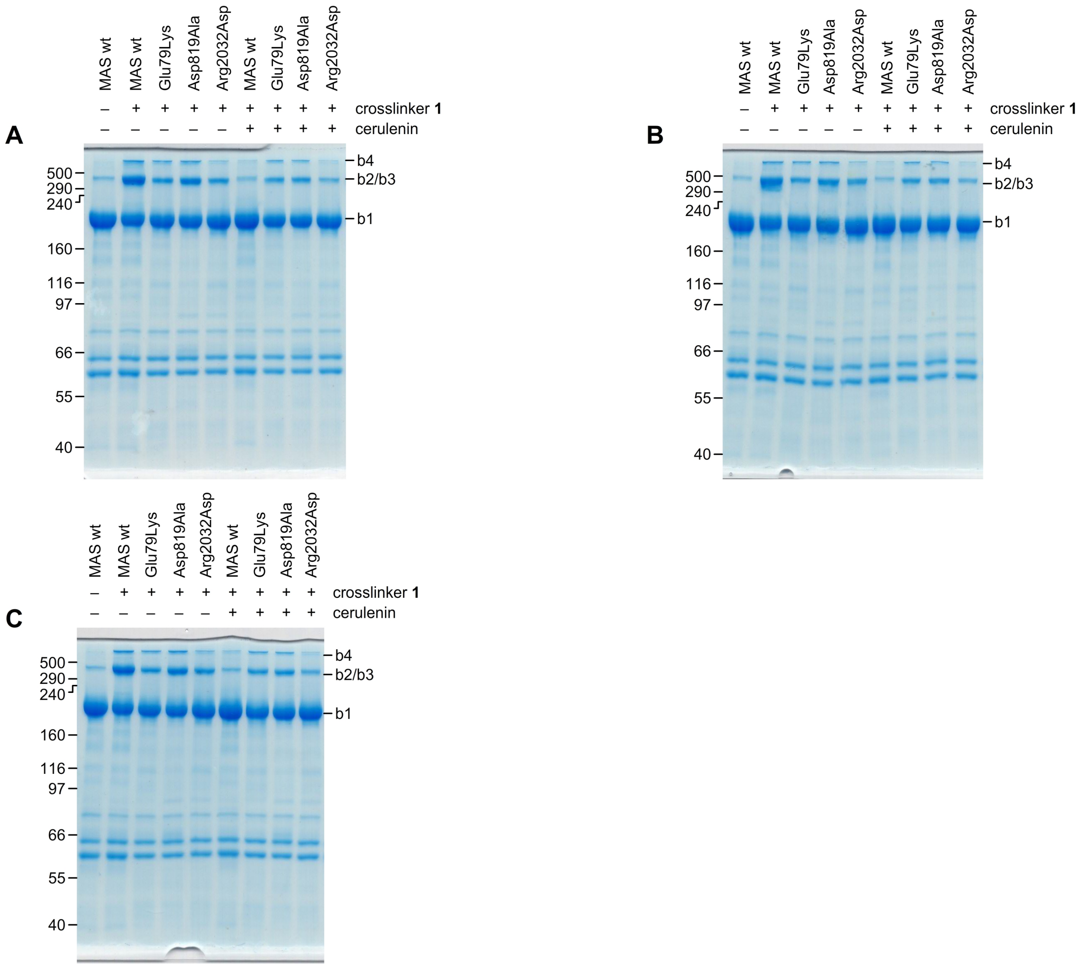

Supplementary Fig. 25 | Crosslinking activity assay SDS-PAGE gels. (**A-C**) Crosslinking activity assay with **1** was performed on MAS wt and MS mutants (Glu79Lys, Asp819Ala and Arg2032Asp). These 6% SDS PAGE gels were used to prepare the data in Supplementary Fig. 22. The crosslinked complexes were identified as four bands, b1, b2, b3, and b4. Complex C, uncrosslinked MAS monomer, and intra-protomerically crosslinked MAS monomer were assigned to b1. Complex B was assigned to b2, and complexes A and D were assigned to b3. Intermolecular crosslinking of MAS (oligomer after denaturing) was observed as b4. A throughout analysis of b1, b2, b3, and b4 can be found in Supplementary Fig. 45.

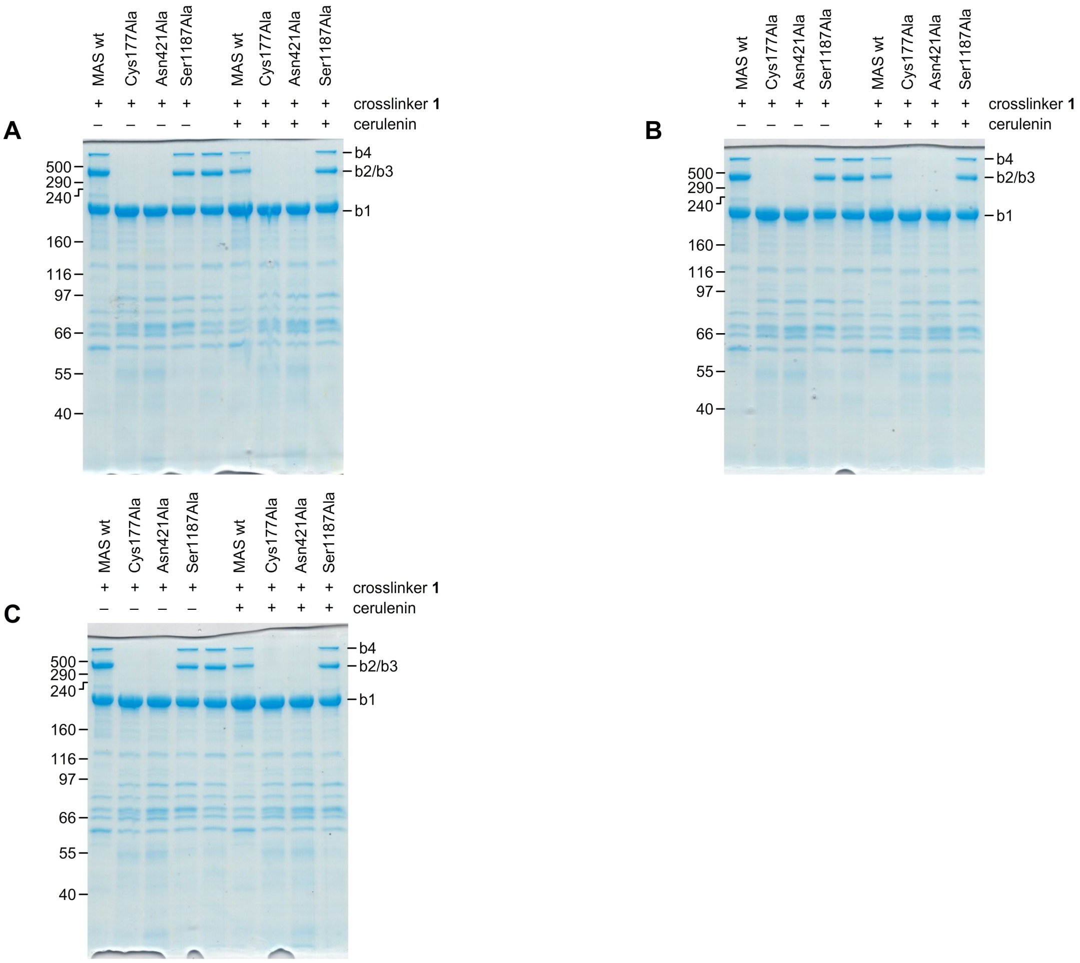

Supplementary Fig. 26 | Crosslinking activity assay SDS-PAGE gels. (**A-C**) Crosslinking activity assay with **1** was performed on MAS wt and MS mutants (Cys177Ala, Asn421Ala and Ser1187Ala). These 6% SDS PAGE gels were used to prepare the data in Supplementary Fig. 22. The crosslinked complexes were identified as four bands, b1, b2, b3, and b4. Complex C, uncrosslinked MAS monomer, and intra-protomerically crosslinked MAS monomer were assigned to b1. Complex B was assigned to b2, and complexes A and D were assigned to b3. Intermolecular crosslinking of MAS (oligomer after denaturing) was observed as b4. A throughout analysis of b1, b2, b3, and b4 can be found in Supplementary Fig. 45.

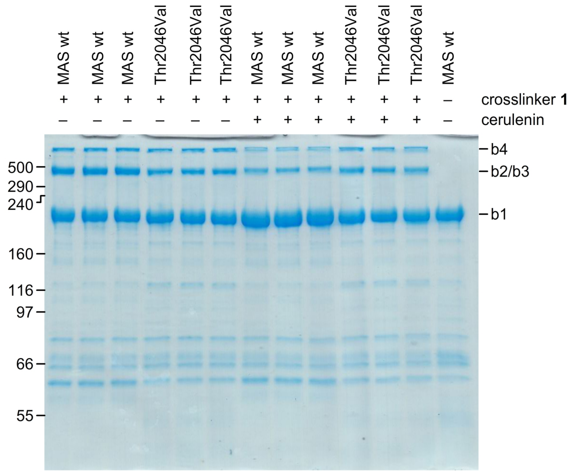

Supplementary Fig. 27 | Crosslinking activity assay SDS-PAGE gels. (**A-C**) Crosslinking activity assay with **1** was performed on MAS wt and MS mutant (Thr2046Val). These 6% SDS PAGE gels were used to prepare the data in Supplementary Fig. 22. The crosslinked complexes were identified as four bands, b1, b2, b3, and b4. Complex C, uncrosslinked MAS monomer, and intra-protomerically crosslinked MAS monomer were assigned to b1. Complex B was assigned to b2, and complexes A and D were assigned to b3. Intermolecular crosslinking of MAS (oligomer after denaturing) was observed as b4. A throughout analysis of b1, b2, b3, and b4 can be found in Supplementary Fig. 45.

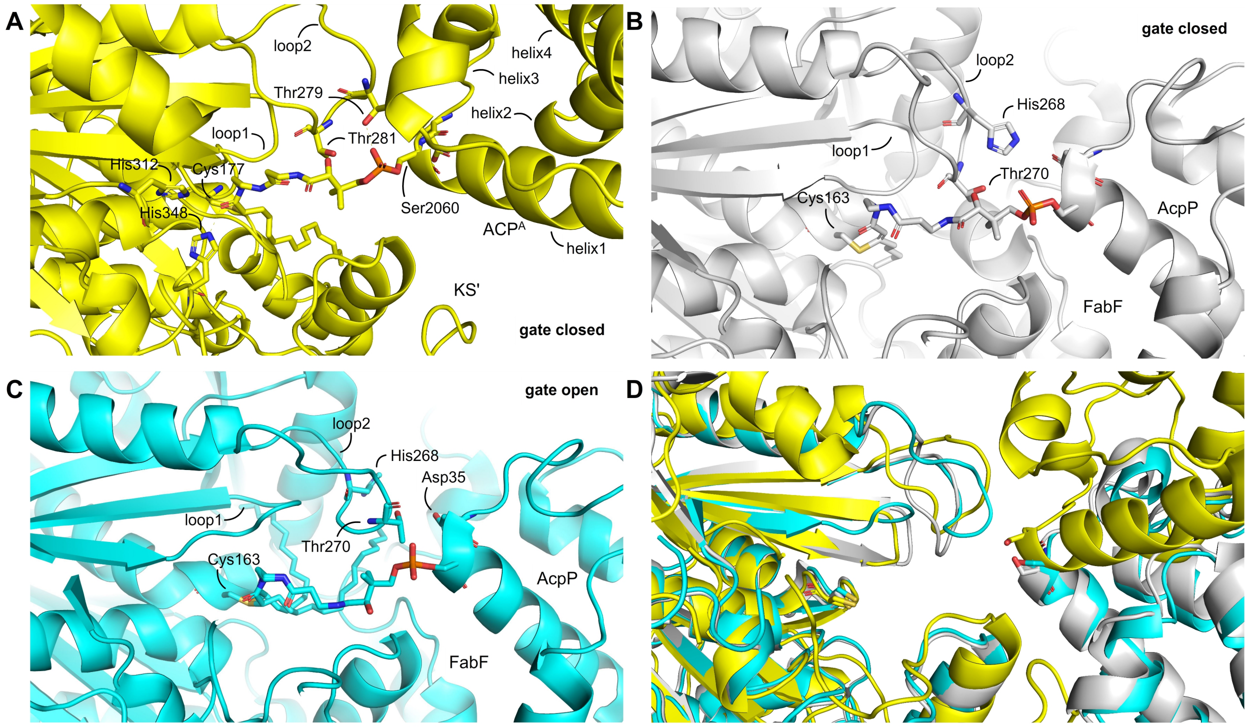

Supplementary Fig. 28 | Comparison of gating loops 1 and 2 between MAS ketosynthases and FabF. (A) ACP^A crosslinked at KS' (complex B) in the gate closed conformation (yellow). Thr279 and Thr281 from gating loop 2 interact with the crosslinker **1** through hydrogen bonding. **(B)** This is similar to the interactions made by Thr270 and His268 in gating loop 2 of FabF from AcpP=FabF with **12** (PDBID: 7L4L). (**C**) In the gate open conformation of AcpP=FabF with **11** (cyan) (PDBID: 6OKG) is regulated by the interaction between AcpP Asp35 and FabF Thr270 while FabF His268 does not make an interaction. (**D**) Alignment of the above structures (without ACP) to the KS dimeric core of complex B (RMSD= 1.959) demonstrates that ketosynthase gating loops 1 and 2 resemble the gate closed conformation in FabF where MAS ketosynthase Thr281 and Thr279 may play a similar role as FabF Thr270 and His268, respectively, in regulating the gating system.

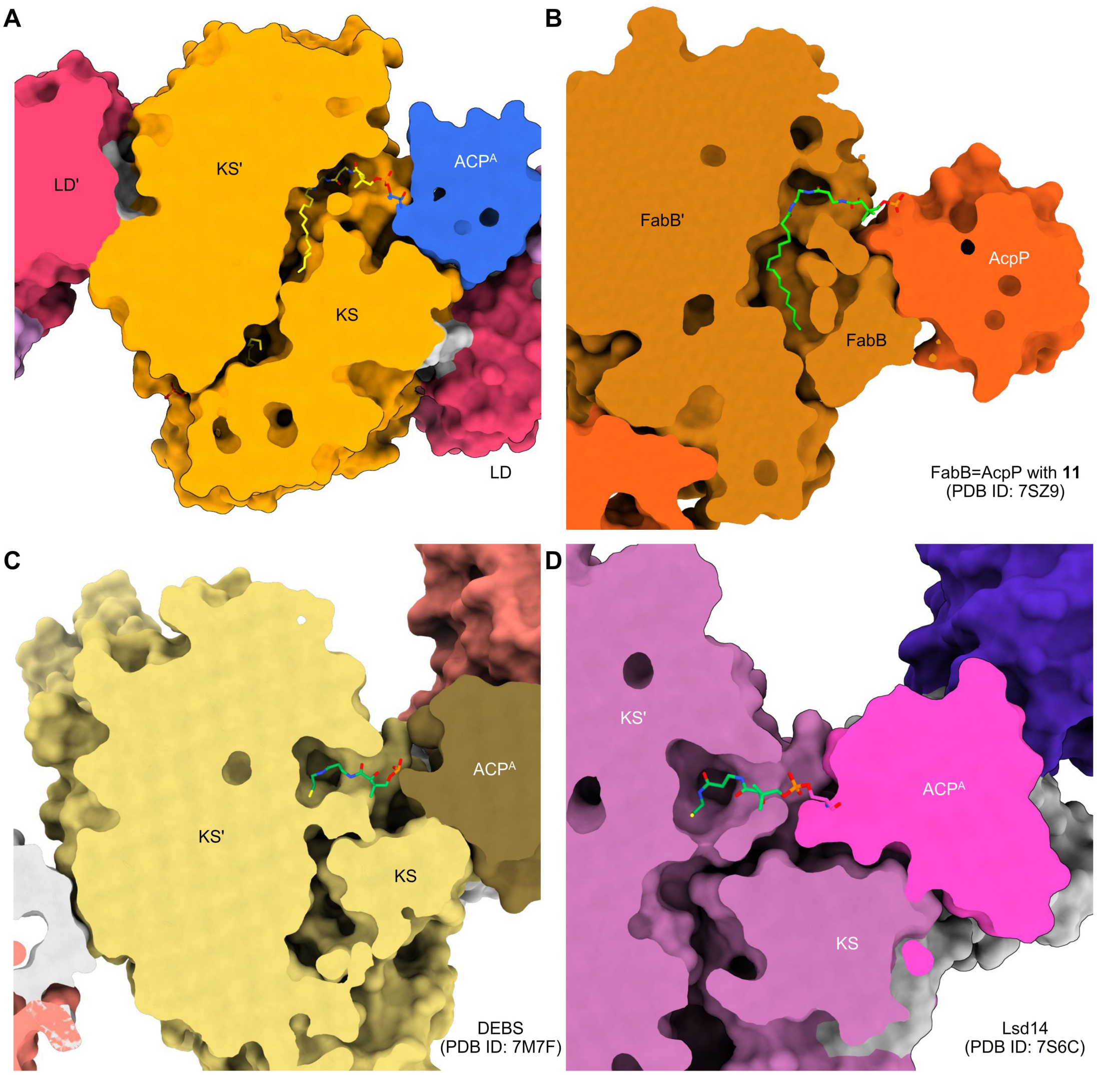

Supplementary Fig. 29 | KS binding tunnel comparison (**A**) Crosslinker **1** (yellow) spans from the ACP (blue) active residue Ser2060 deep into the binding pocket formed by KS • KS' dimer (orange) (complex B). The two narrow but long binding tunnels formed by the dimeric KS • KS' core are pointing at each other and connected (the connection is beneath the surface shown). (**B**) Crosslinker **1** (green) is buried deep inside the more spacious *E. coli* KS FabB (brown) binding tunnel. The crosslinker points in a different direction when compared to the MAS ketosynthase. It is worth noting that the two binding tunnels of FabB are not connected. A negative cooperativity between these two tunnels is governed by the E200-Q113 back gate residing at the end of the tunnels. Such negative cooperativity can regulate the production of both medium and long chain fatty acids in FabB. (**C**) DEBS-M1 KS (yellow) adopts a narrow entrance of the binding tunnel for the pantetheine arm. As the native substrates of DEBS KS is partially reduced (not straight chain fatty acid), the second half of the binding tunnel becomes relatively more spacious.[13] (**D**) LSD14 KS (purple) adopts a larger binding tunnel without a well-defined end.[14]

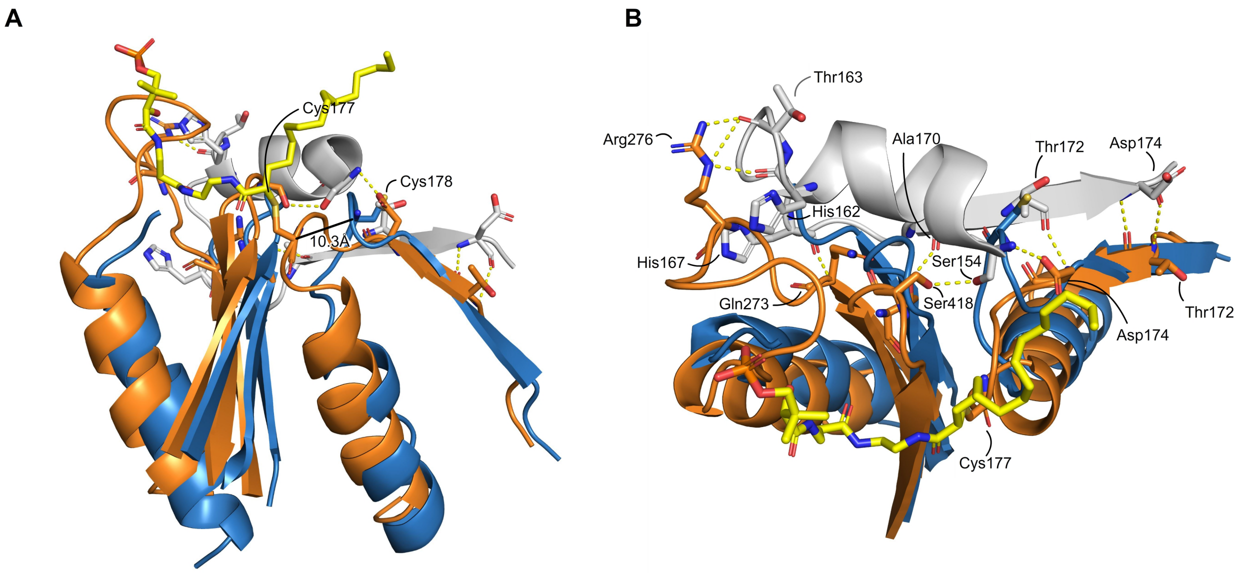

Supplementary Fig. 30 | KS • KS' dimerization interface stabilizes ketosynthase active sites. (A) Alignment of MAS-like PKS KS monomer (PDBID: 5BP1) to complex B (RMSD= 0.623) demonstrated the overlay of the ketosynthase active sites of MAS (orange for KS' and grey for KS) from complex B and MAS-like PKS KS (ocean blue). Compared with the active residue Cys177 of MAS complex B, MAS-like PKS KS-LD-AT tridomain crystalized as a monomeric form demonstrates the active site residue Cys178 was observed 10.2 Å away. This positioning was reported to be catalytically inactive.[5] **(B)** Demonstration of the dimerization interface that the MAS-like PKS structure stabilizes the KS reactive site in MAS complex B.

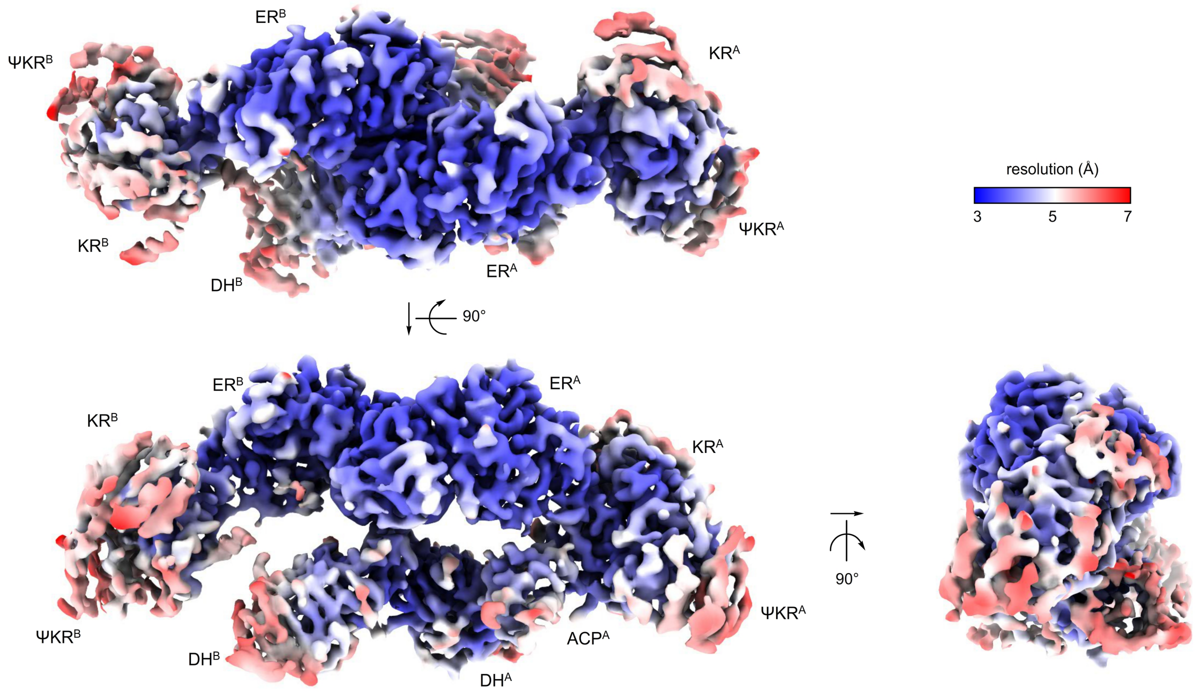

Supplementary Fig. 31 | Density Map of MAS complex C. Cryo-EM map of MAS complex C with ACP crosslinked to DH at a contour level of 0.19. Similar to what was observed for complexes A and B, protomer (DH-ΨKR-ER-KR) with ACP crosslinked demonstrates a higher resolution than the protomer without the other ACP crosslinked.

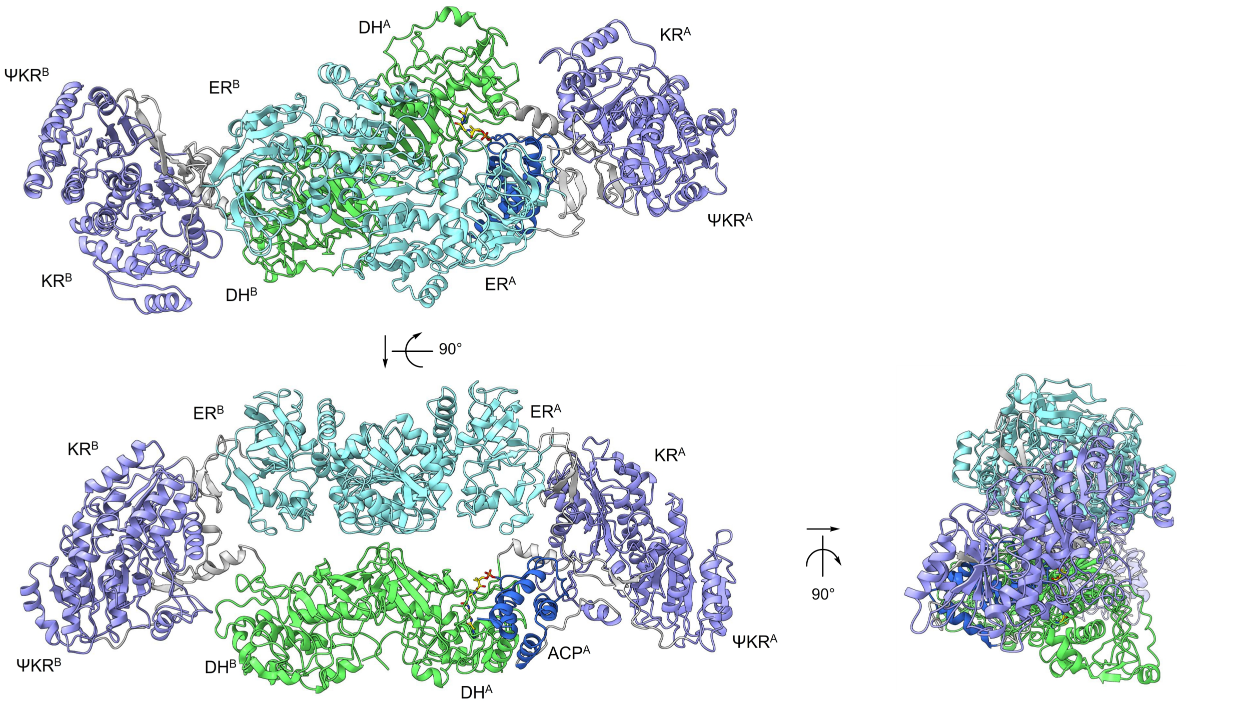

Supplementary Fig. 32 | Architecture of MAS complex C. Front, top, and side views of MAS complex C which consists of DH (green), ΨKR (purple), ER (light blue) and KR (purple) with ACP (blue) crosslinked at DH through **1** (yellow).

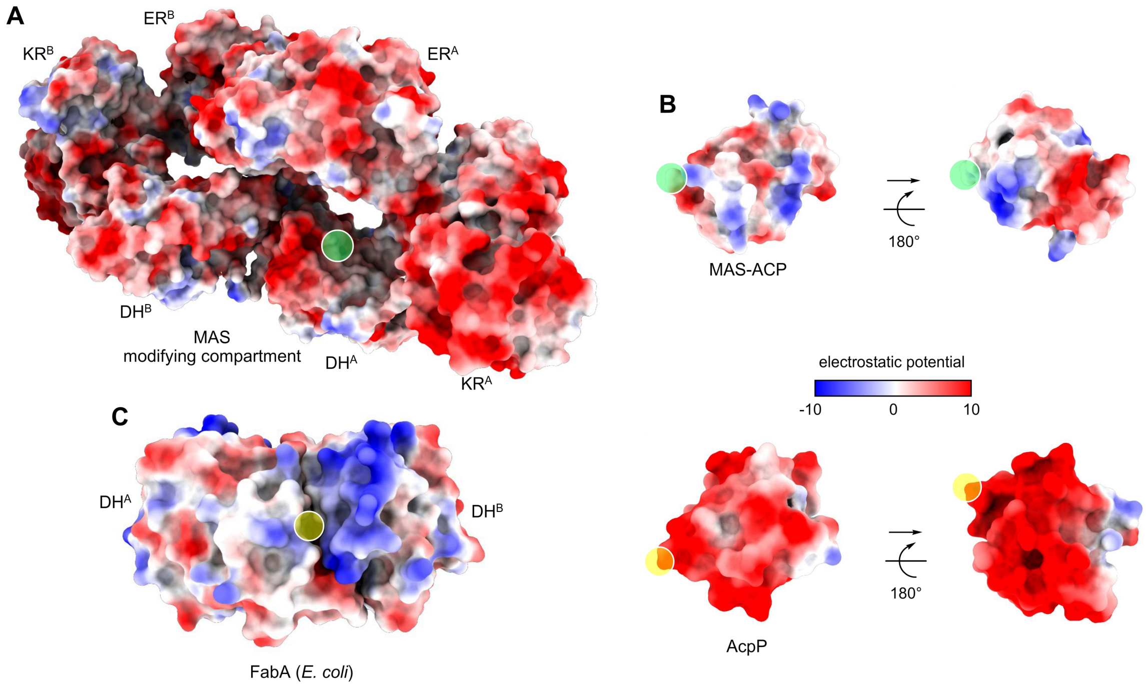

Supplementary Fig. 33 | Electrostatic profiles of MAS modifying compartment and FabA. (**A**) The electrostatic potential profile of MAS modifying compartment (DH-ΨKR-ER-KR) from complex C with the entrance of the DH binding tunnel highlighted by a green dot. DH binding site reveals a moderately negative charged interface. (**B**) Electrostatic potential profile of MAS ACP with its active residue Ser 2060 highlighted by a green dot. (**C**) When compared with MAS DH, *E. coli* Type II DH FabA (PDBID: 4KEH) possesses a positively charged AcpP binding interface which is opposed to the negatively charged AcpP.

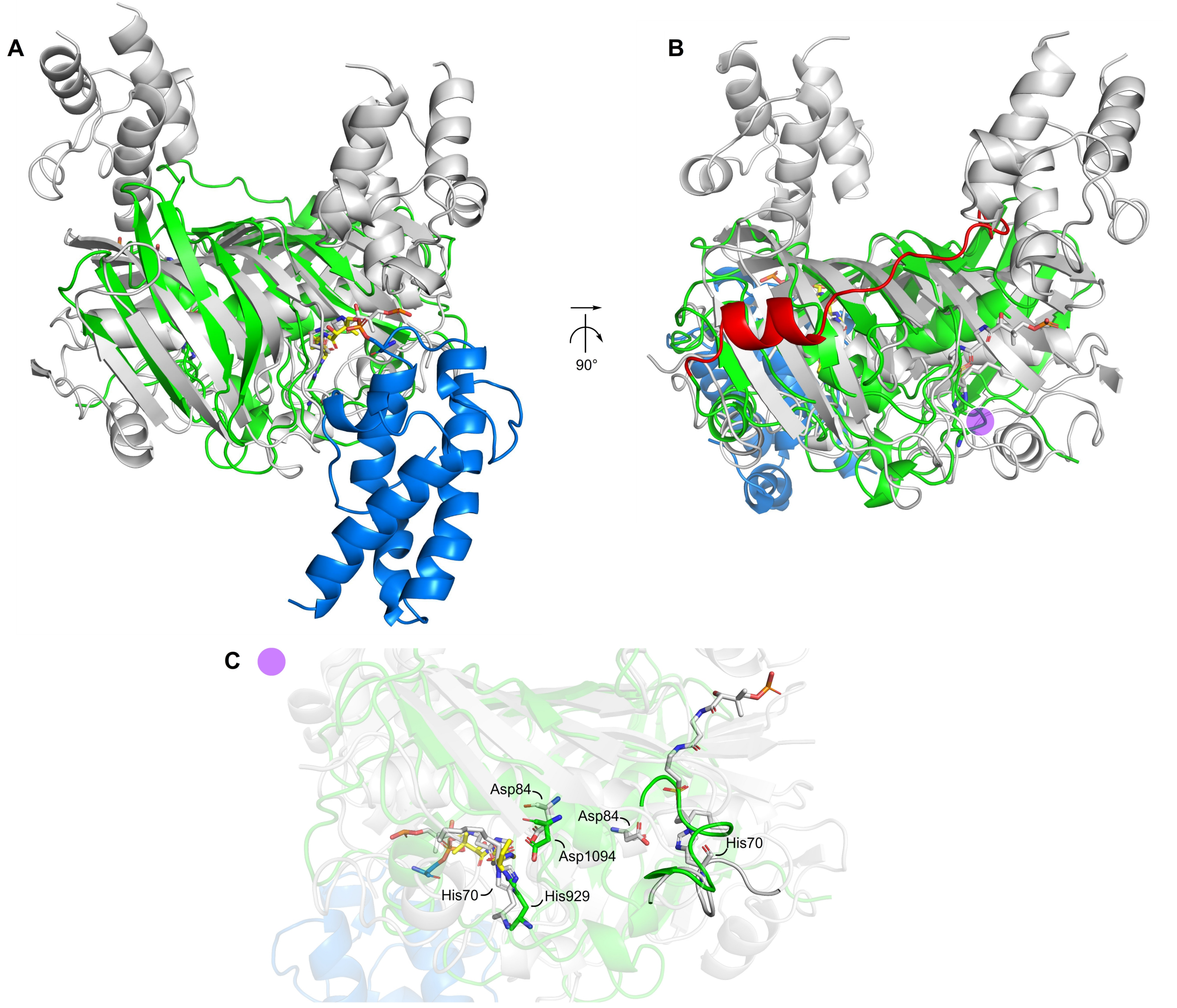

Supplementary Fig. 34 | Comparison between DH and FabA. Overlay of MAS ACP (blue) crosslinked with MAS DH (green) from complex C through **1** (yellow) and *E. coli* type II AcpP (grey) crosslinked with DH FabA (grey) (PDBID: 4KEH) with **10**.[38] (**A**) Front view illustrates the orientation difference of MAS ACP and AcpP binding. Despite this difference, both substrates use similar binding tunnels. (**B**) Back view of the previous overlay. Instead of forming a homodimer like FabA, MAS DH consists of a double-hotdog fold linked by a 20 aa long loop (red). (**C**) Unlike the homodimeric FabA, the double-hotdog fold in MAS DH is composed of only one set of catalytic residues (His929 and Asp1095). Although one of the hotdog folds does not have reactive residues, it plays an important role in forming dimerization interface with the other DH to facilitate the dimerization of the MAS architecture.

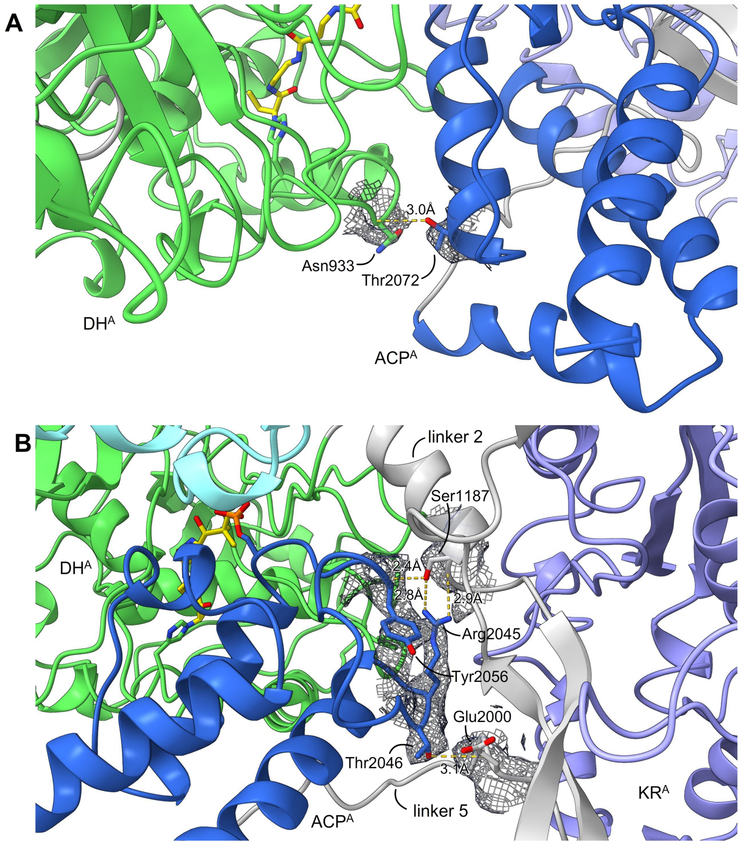

Supplementary Fig. 35 | ACP interactions in complex C. At a contour level of 0.19, the density of (**A**) Thr2072 in ACP forms hydrogen bond with Asn933. (**B**) Ser1187 and Glu2000 from linkers 2 and 5, respectively, interact with Arg2045, Tyr2056 and Thr2046 from ACP in complex C, emphasizing the important interactable features of linkers in MAS.

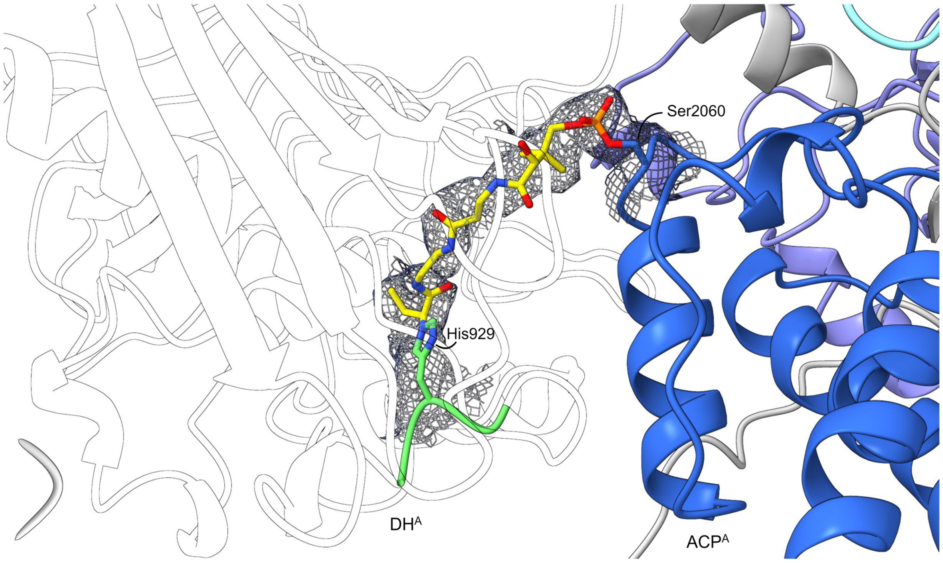

Supplementary Fig. 36 | Visualization of crosslinker 1 in complex C. A close-up depiction of the crosslinking between ACP (blue) and DH (transparent with silhouette) from complex C. Crosslinker **1** (yellow) forms a phospho-linkage at Ser2060 of ACP and a covalent linkage at the DH active residue His929 (green). Continuous density (contour level= 0.12) was observed up to the δ carbon of the crosslinker including the covalent linkages between ACP and DH.

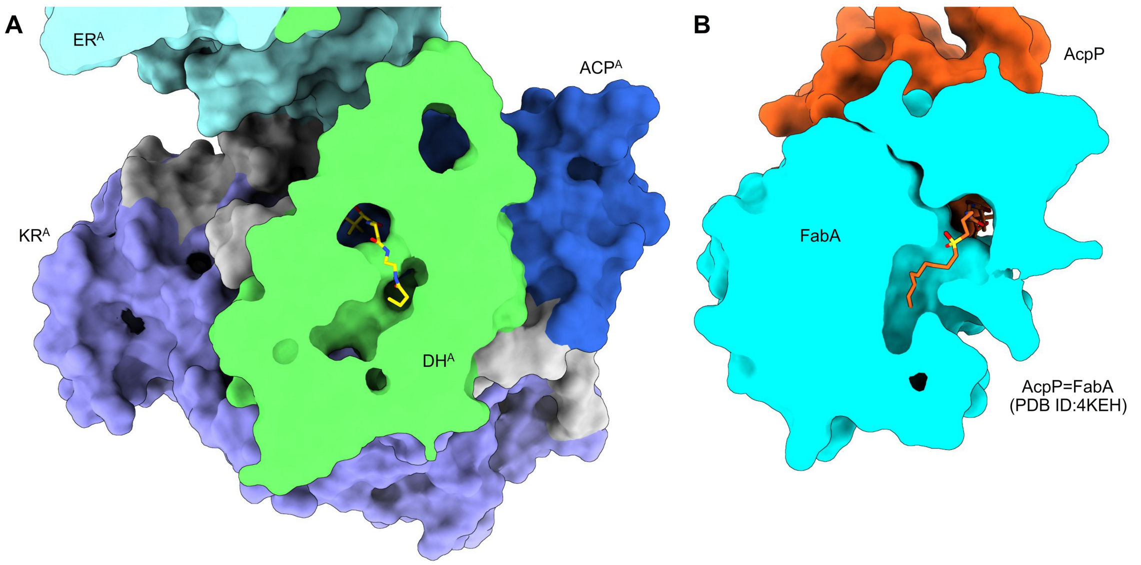

Supplementary Fig. 37 | Binding tunnels comparison between DH and FabA. (**A**) DH (green) from complex C contains a deep binding tunnel for its natural substrate β-hydroxy mycocerosic acids. The substrate in this figure is **1**, although continuous map density supports up to the δ carbon. (**B**) *E. coli* Type II DH FabA (sky blue) contains a finite and shorter tunnel for its preferred natural substrate β-capric acid. The crosslinker in the figure is DH targeting **10** (Figure 3). [38]

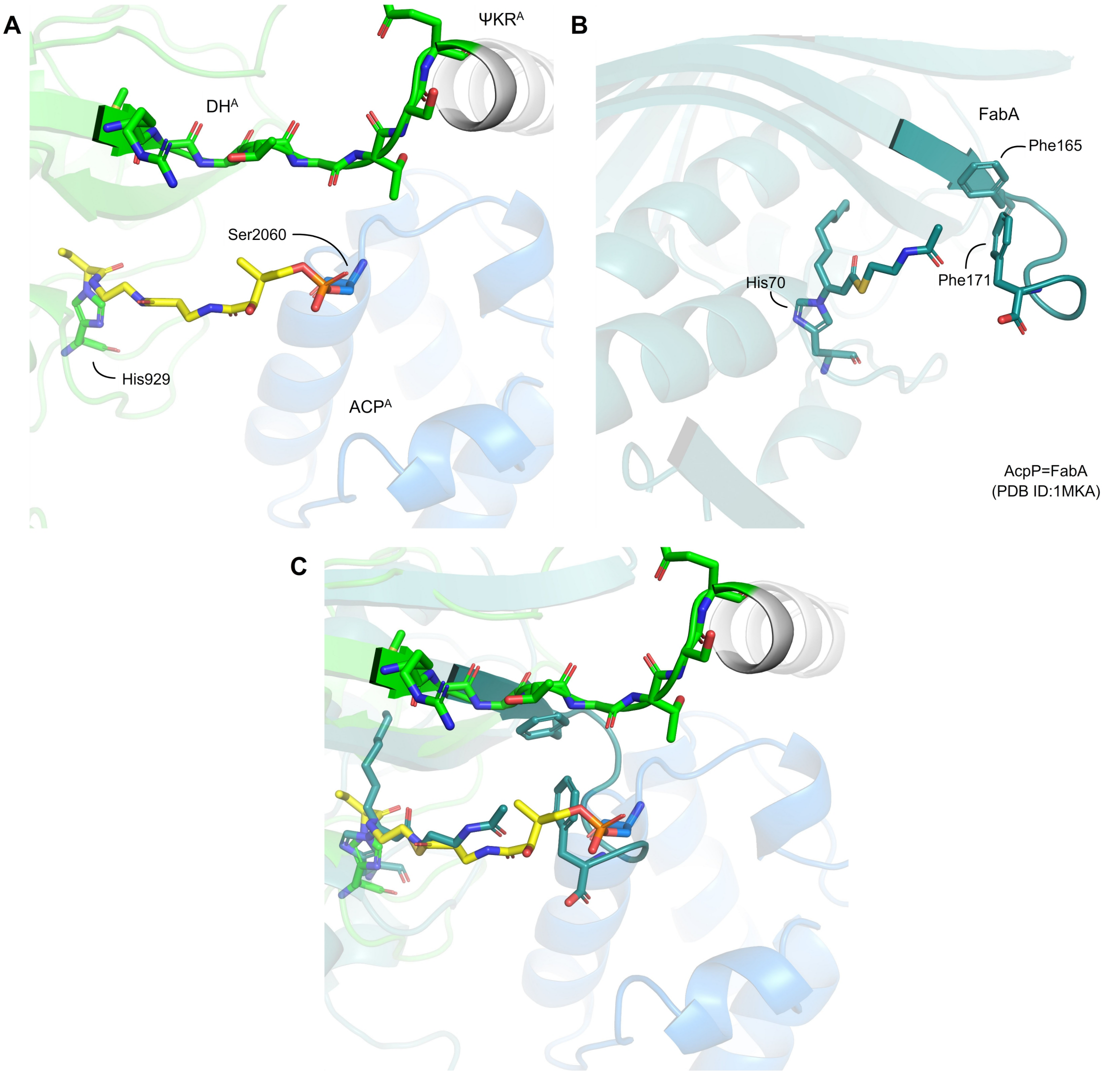

Supplementary Fig. 38 | DH lacks gating residues in FabA. (**A**) The C terminal of DH connects to ΨKR lacking (**B**) gating residues Phe165 and Phe171 in *E. coli* Type II DH FabA. (**C**) Overlay of the DH-ACP from complex C and FabA (PDBID: 1MKA).[42]

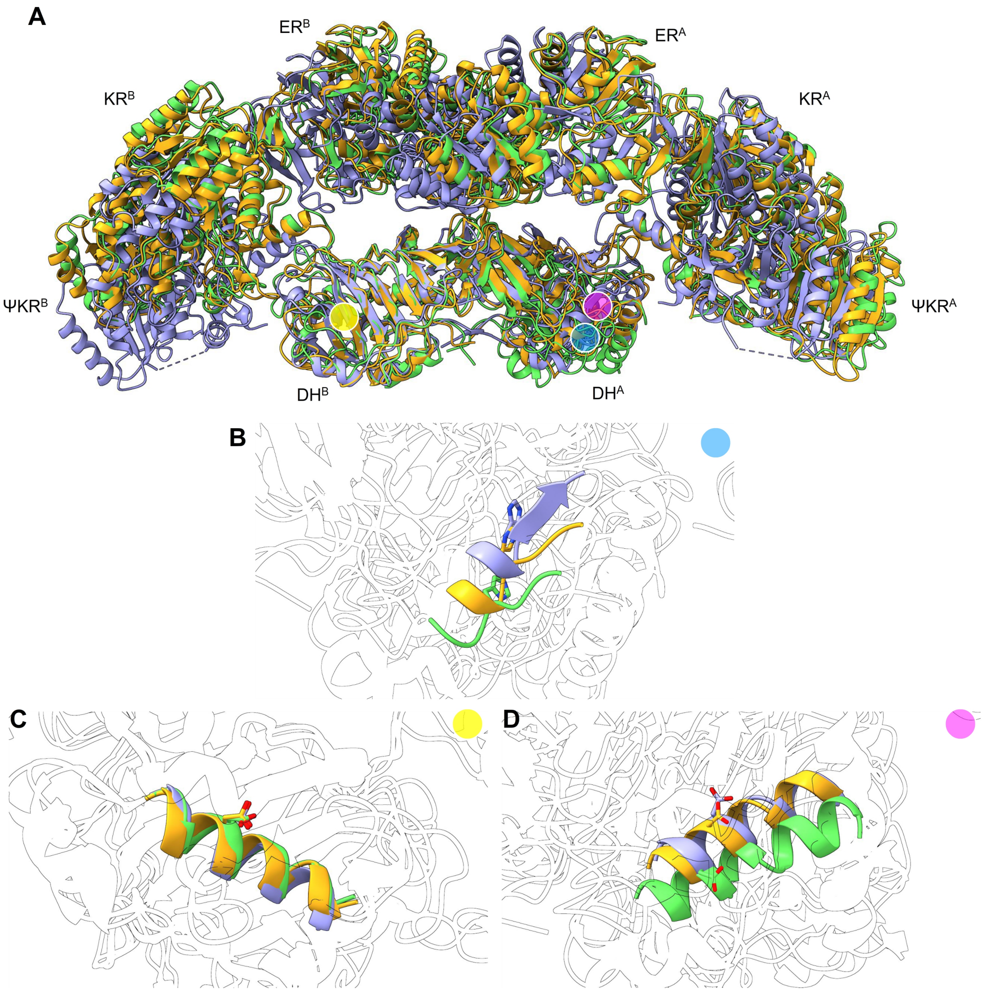

Supplementary Fig. 39 | DH rotation upon ACP crosslinking. (**A**) Overlay of complex C (ACP not shown for simplicity) crosslinked at DH (green), complex A with *apo*-DH (orange), and MAS-like PKS modifying compartment crystal structure with *apo*-DH (purple, PDB: 5BP4) (RMSD= 1.568). While the structures were aligned to the *apo*-DH of complex C, significant DH rotation and translation were observed (light blue dot). (**B**) Complex C with ACP crosslinked to DH, the reactive residue His929 rotates outward along with the DH rotation to be more accessible for ACP binding. In contrast, His929 of complex A *apo*-DH (orange) or His934 of MAS-like PKS (purple) points more inward at KR becoming less accessible for ACP binding due to steric occlusion. (**C**) The rotation and translation movement of DH can also be observed on the reactive residue Asp residing on an α-helix (amino acids 1092-1102 for complex A in orange as well as complex C in green and 1096-1107 for MAS-like PKS in purple). While there is no significant movement in *apo*-DHs without ACP binding, (**D**) ACP crosslinked DH in complex C translated and rotated outward. Such ACP binding dependent rotation is possible to serve for the regulation of substrate or ACP binding. ACP and **1** are not shown in this figure for simplicity.

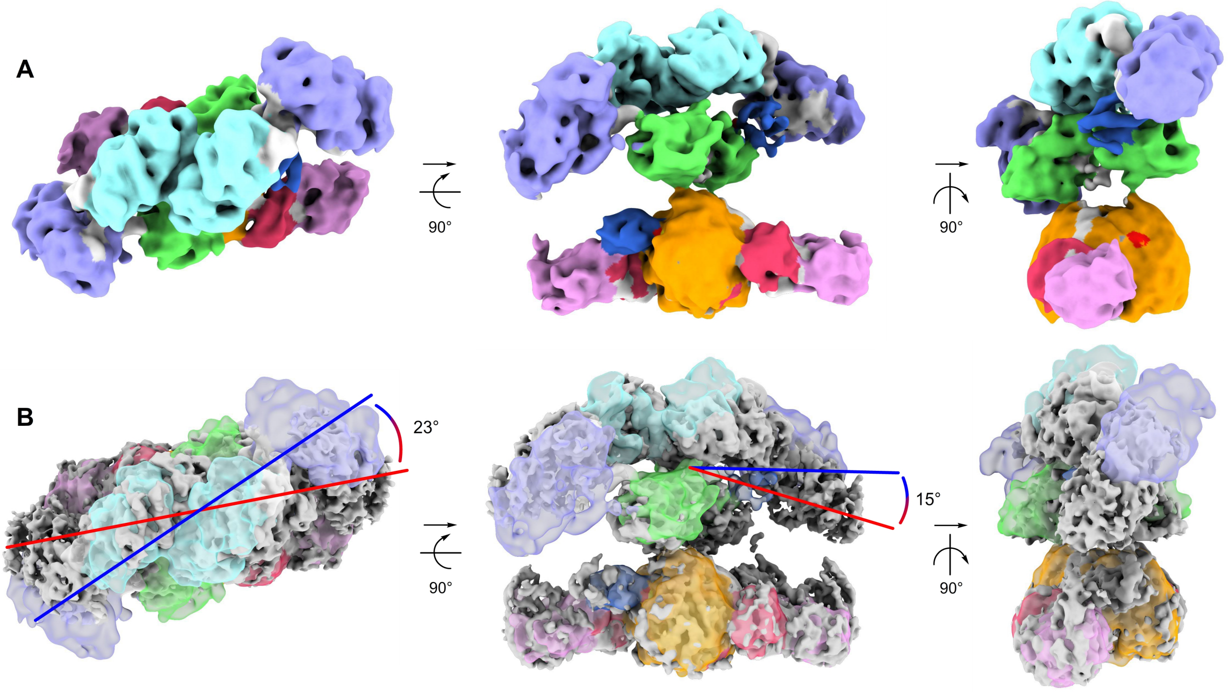

Supplementary Fig. 40 | MAS complex D exemplifies higher order dynamics. (**A**) Front, side, and top views of the density map of complex D (contour level of 0.058) with each domain colored according to Fig. 2. The structure was not modeled into this map due to the modest resolution (6.18 Å). This map visualized both ACPs trapped at DH and KS, respectively. MAS can operate synchronously where both ACPs can work at the same time in catalytic chambers 1 and 2. It also revealed higher-order dynamics showcasing that both catalytic chambers can process substrates asymmetrically. Here, ACP visits a domain in catalytic chamber 1 that is different from the domain that the other ACP visits in chamber 2. (**B**) Overlay of the density maps of complex D (colored and transparent) and complex A (grey and solid) demonstrating a 23° twist and 15° tilt of the modifying compartment.

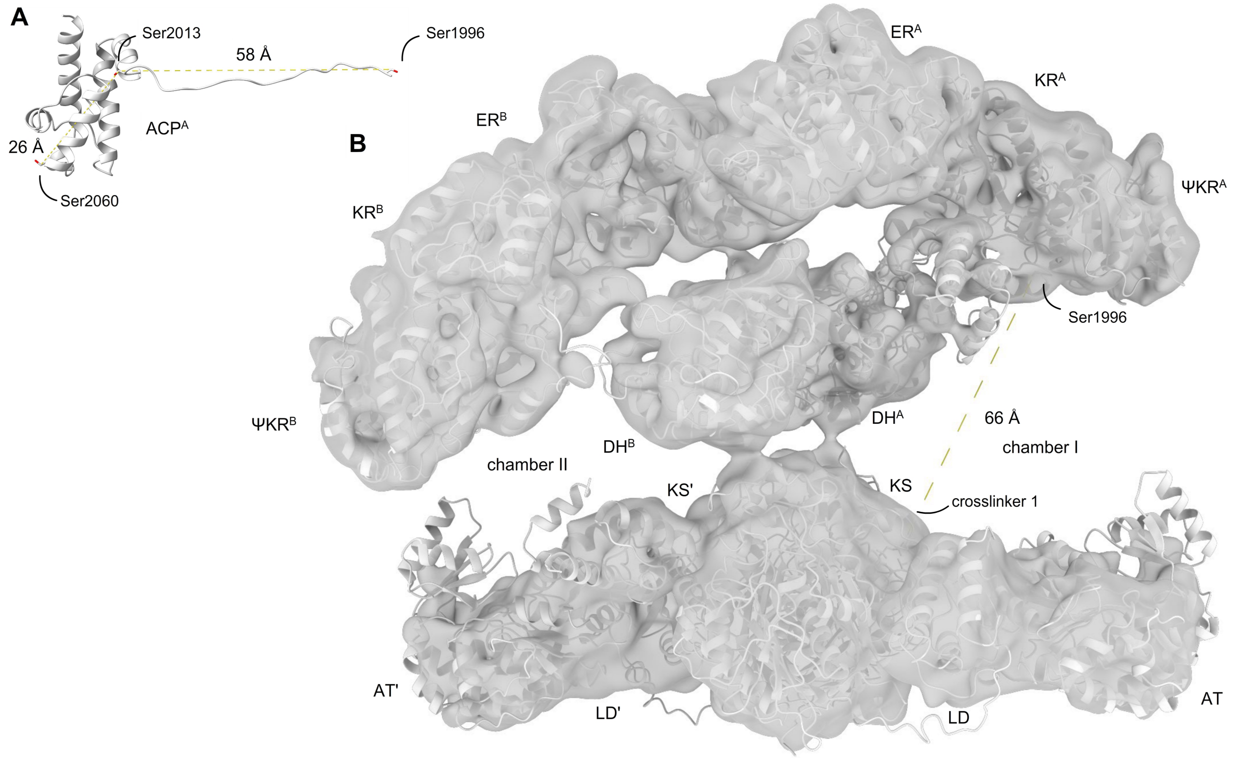

Supplementary Fig. 41 | ACP travel distance. (**A**) Estimation of the allowed travel distance of ACP^A as measured from Cβ of Ser1996 (N-terminus of linker 5) to Cβ of Ser2013 (ACP) to Cβ of Ser2060 (ACP reactive residue). **(B)** Density map of complex D fitted with the models of complexes B and C demonstrating the distance between Cβ of Ser1996 (N-terminus of linker 5) to the phosphorus of crosslinker **1** at the entrance of the KS binding tunnel.

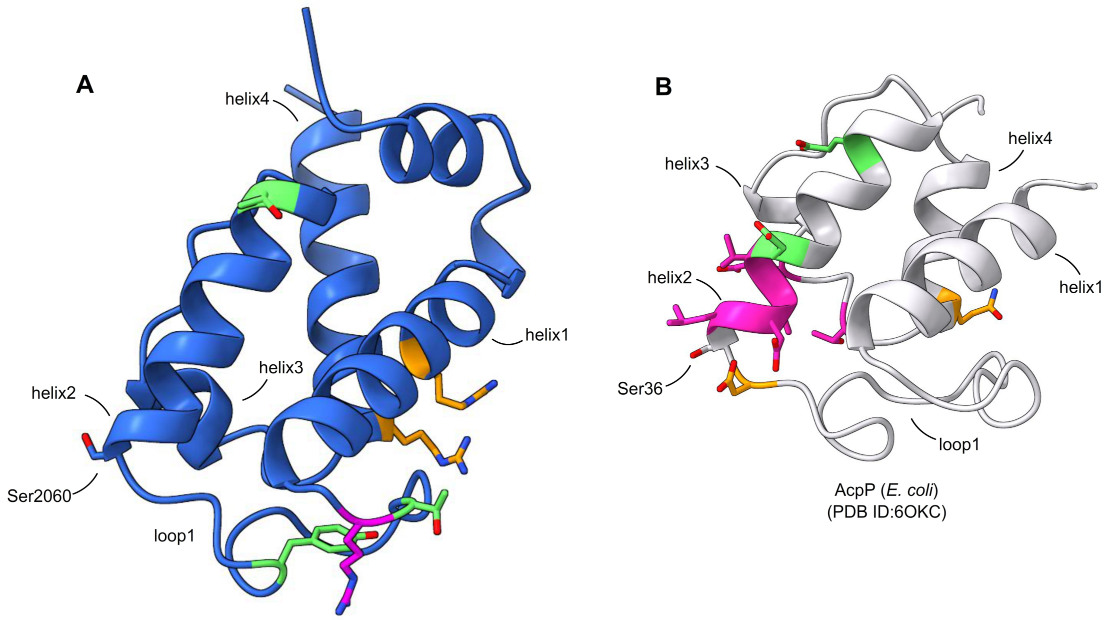

Supplementary Fig. 42 | Simplified view of the interactive residues in MAS ACP and *E. coli* AcpP. (**A**) ACP consists of helices 1-4. Residues in orange and purple are responsible for interactions with KS • KS'. Residues in green and purple are responsible for interactions with DH. (**B**) *E. coli* Type II AcpP structure consists of helices 1-4 and loop 1. Residues in orange and purple are responsible for interactions with type II KSs FabF and FabB. Residues in green and purple are responsible for interactions with Type II DHs FabA and FabZ.[65]

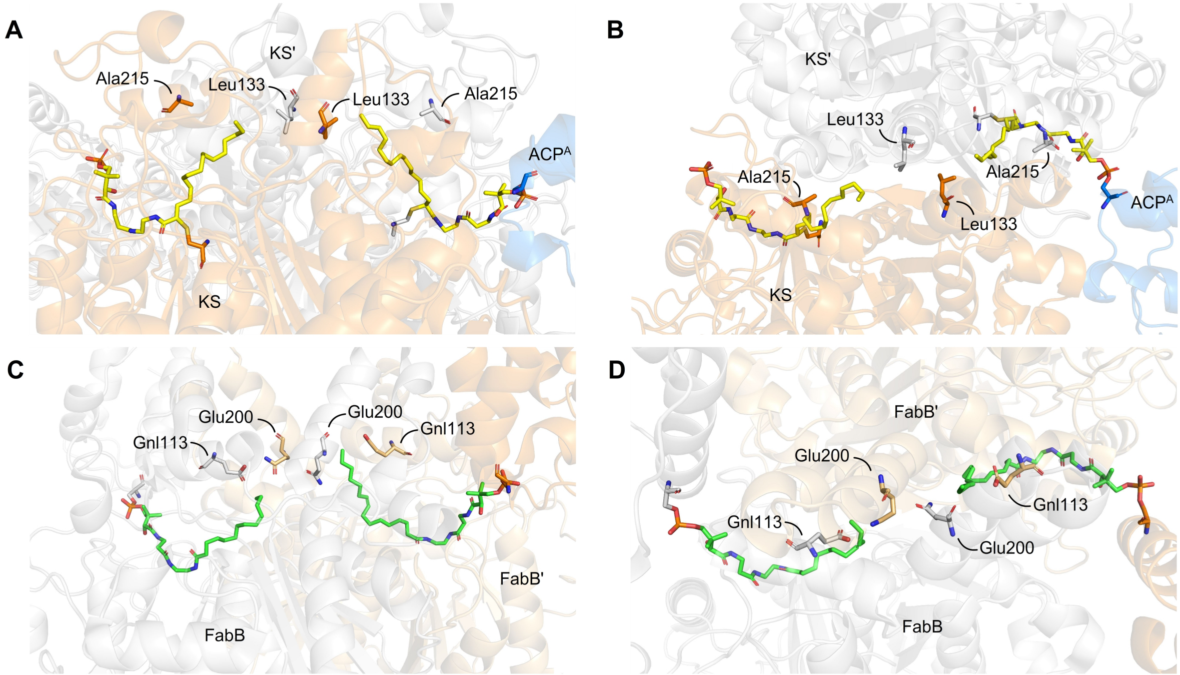

Supplementary Fig. 43 | The connection between the two KS binding tunnels. (**A**) Side and (**B**) top views of MAS KS • KS' (orange and grey) from complex B demonstrate the connection between the two MAS binding tunnels with **1** (yellow). (**C**) Side and (**D**) top views of FabB KS • KS' (light gold and grey) demonstrate the connection between the two FabB binding tunnels with **11**. The negative cooperativity of FabB regulates the substrate binding specificity by controlling the "back gate" through the hydrogen bonding within the two sets of Glu200 and Gln113. In MAS KS, it does not have a "back gate".

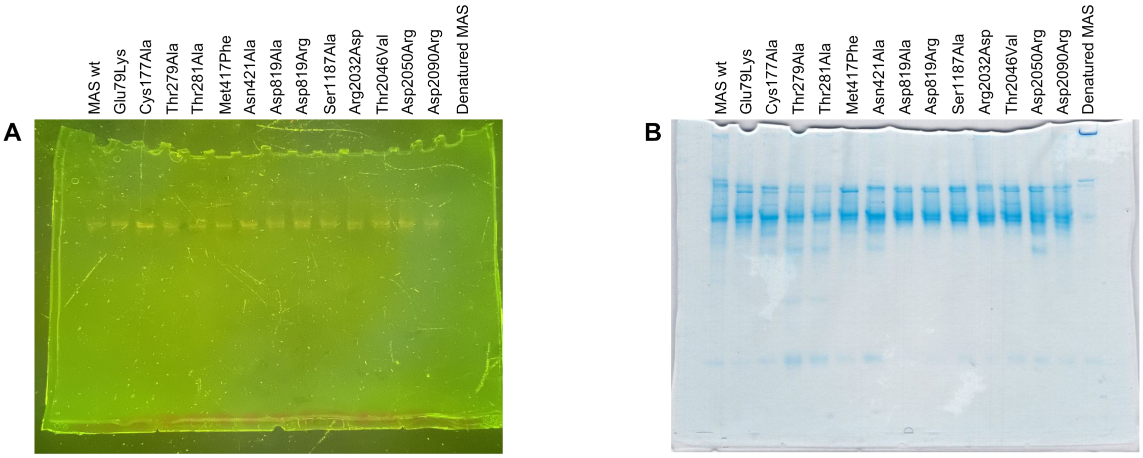

Supplementary Fig. 44 | Analysis of MAS and MAS mutant folding. (**A**) Fluorescent labeling of MAS, denatured MAS, and 13 MAS mutants with **13**. Probe **13** (Supplementary Fig. 3) can be loaded onto active site residue Ser2060 of ACP by Sfp, therefore it can serve as an evaluation for the misfolding in MAS and MAS mutants. All proteins were labeled excepted for denatured MAS (prepared by boiling for 5 min). The fluorescent signal was evaluated on a 4-15% native gel with a UV transilluminator at 365 nm (fluorescent) and stained with Coomassie Brilliant Blue (total protein). (**B**) After Coomassie Brilliant Blue staining, the native gel shows MAS and MAS mutants share similar electrophoretic mobility. A different mobility was observed for denatured MAS.

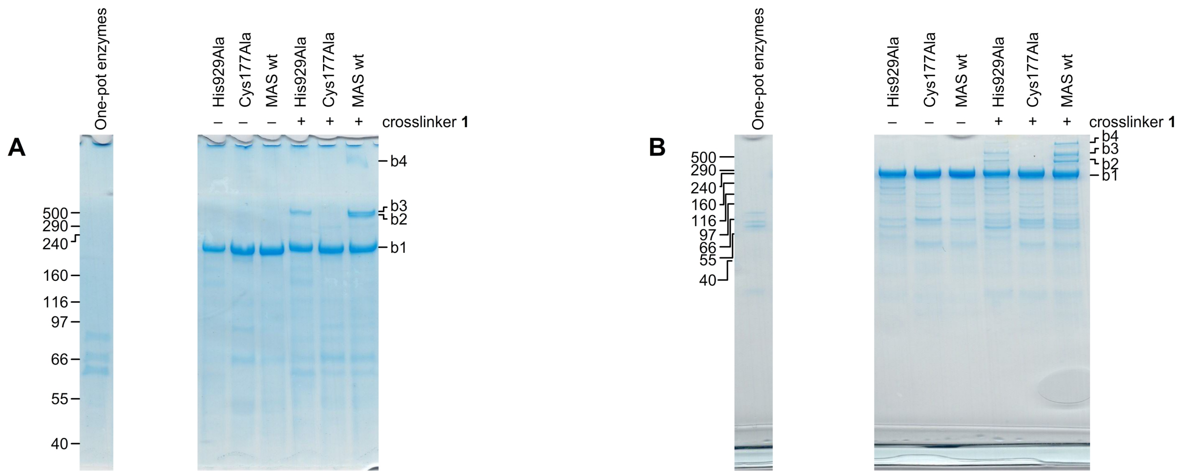

Supplementary Fig. 45 | Identifying crosslinked MAS complexes on SDS-PAGE. As crosslinking in complexes A, B and D produces dimers even after denaturing, they have a higher molecular weight visualized by bands b2 and b3 on: (**A**) 6% SDS-PAGE gel (Supplementary Fig. 2). After denaturing, the conformational difference of the doubly crosslinked complexes A and D are likely to travel slower on gel. Similar observation has been made in studies on FAS.[66] Based on this, b2 was assigned as complex B and b3 as complexes A and D. Since complex C is a monomer after denaturing, along with uncrosslinked MAS monomer and intra-protomerically crosslinked MAS monomer, they were assigned to b1. Minor amount of intermolecular crosslinking of MAS (oligomer after denaturing) was observed as b4 with the highest molecular weight. When the complexes are run on (**B**) 4 to 12% NuPAGE Bis-Tris Mini Protein Gel, bands b2 and b3 were well separated. KS and DH active site residue mutants Cys177Ala and His929Ala mutants were evaluated to confirm these assignments. In Cys177Ala, only crosslinking between ACP and DH can take place, therefore b2, b3, and b4 cannot be observed. On the other hand, a significantly lower crosslinking yield for both b3 and b4 was observed for His929Ala indicating the correct band assignment for the complexes.

A

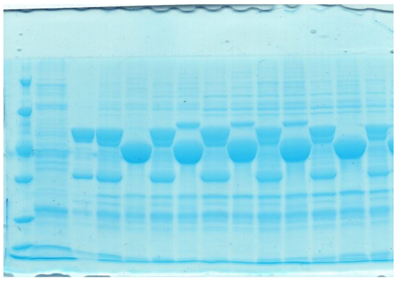

Supplementary Fig. 5 Panel A

B

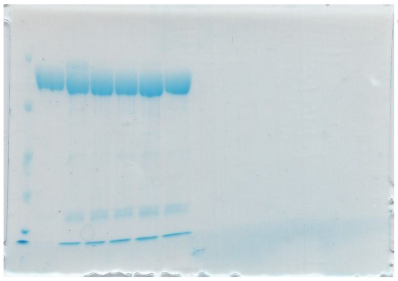

Supplementary Fig. 5 Panel B

C

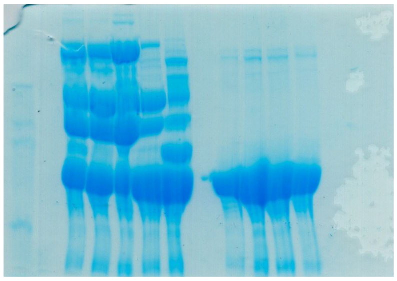

Supplementary Fig. 5 Panel C

D

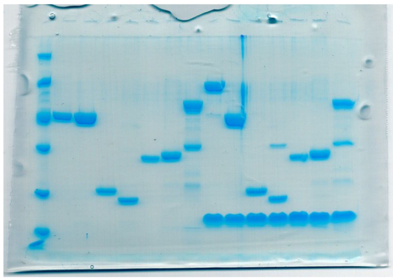

Supplementary Fig. 6 Panel A

E

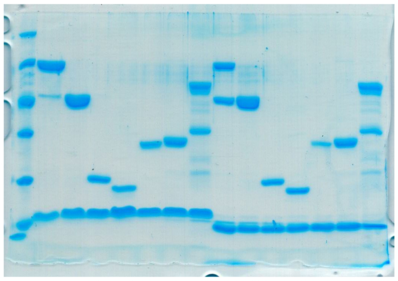

Supplementary Fig. 6 Panel B

F

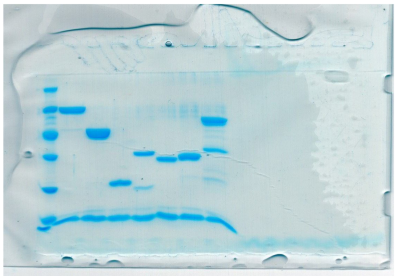

Supplementary Fig. 6 Panel C

G

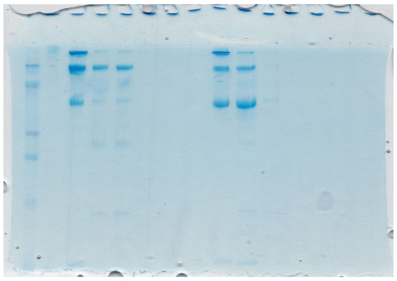

Supplementary Fig. 7

H

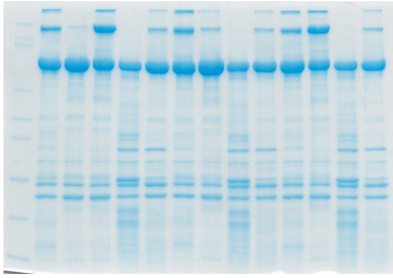

Supplementary Fig. 23 Panel a

I

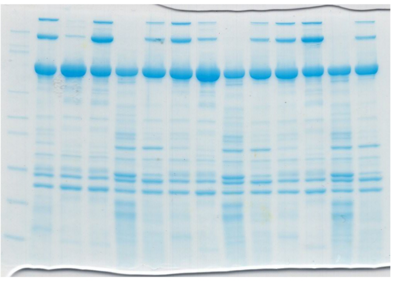

Supplementary Fig. 23 Panel b

J

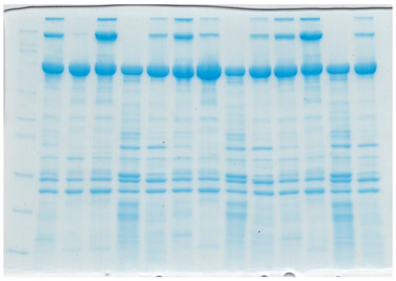

Supplementary Fig. 23 Panel c

K

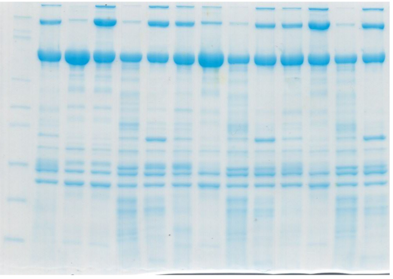

Supplementary Fig. 24 Panel a

L

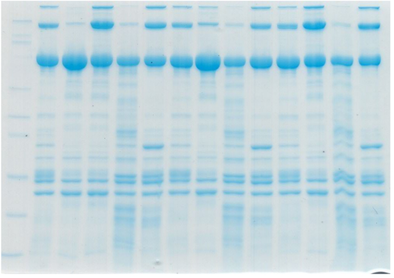

Supplementary Fig. 24 Panel b

M
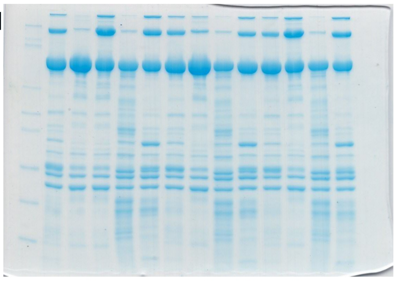
Supplementary Fig. 24 Panel c

N
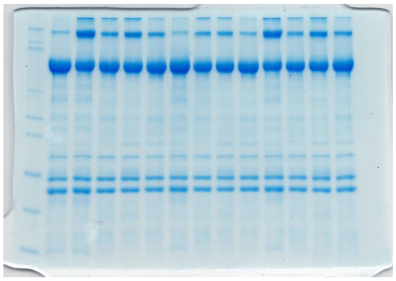
Supplementary Fig. 25 Panel a

O
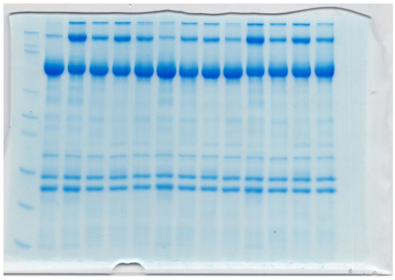
Supplementary Fig. 25 Panel b

P
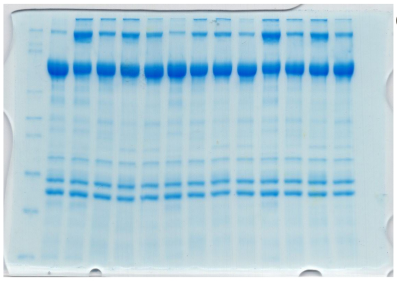
Supplementary Fig. 25 Panel c

Q
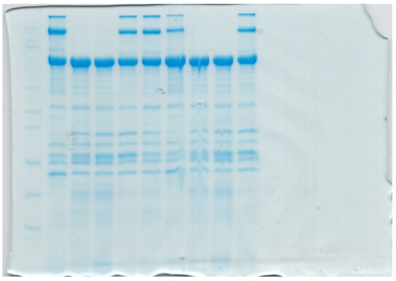
Supplementary Fig. 26 Panel a

R
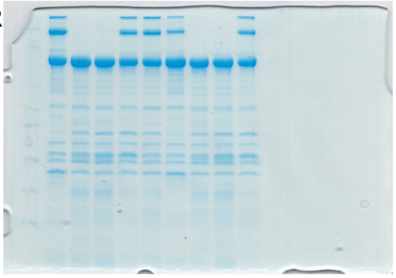
Supplementary Fig. 26 Panel b

S
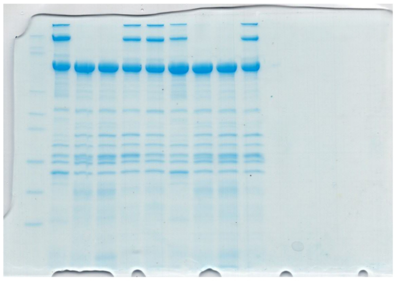
Supplementary Fig. 26 Panel c

T
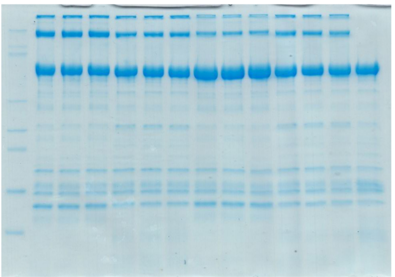
Supplementary Fig. 27

U
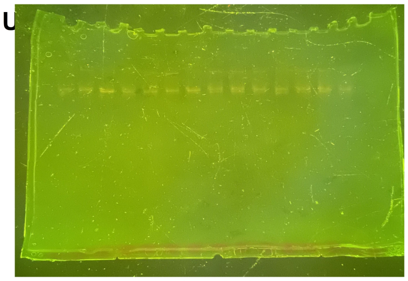
Supplementary Fig. 44 Panel b

V
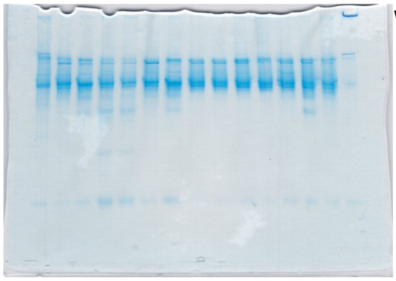
Supplementary Fig. 44 Panel b

W
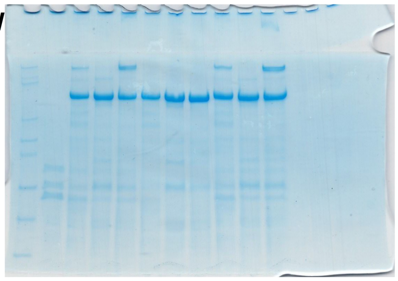
Supplementary Fig. 45 Panel a

X
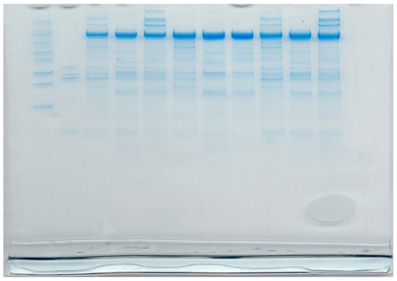
Supplementary Fig. 45 Panel b

Supplementary Fig. 46 | Raw images of SDS PAGE gels. Full size images are provided for gel images cropped, including SDS page gel presented in within this manuscript.

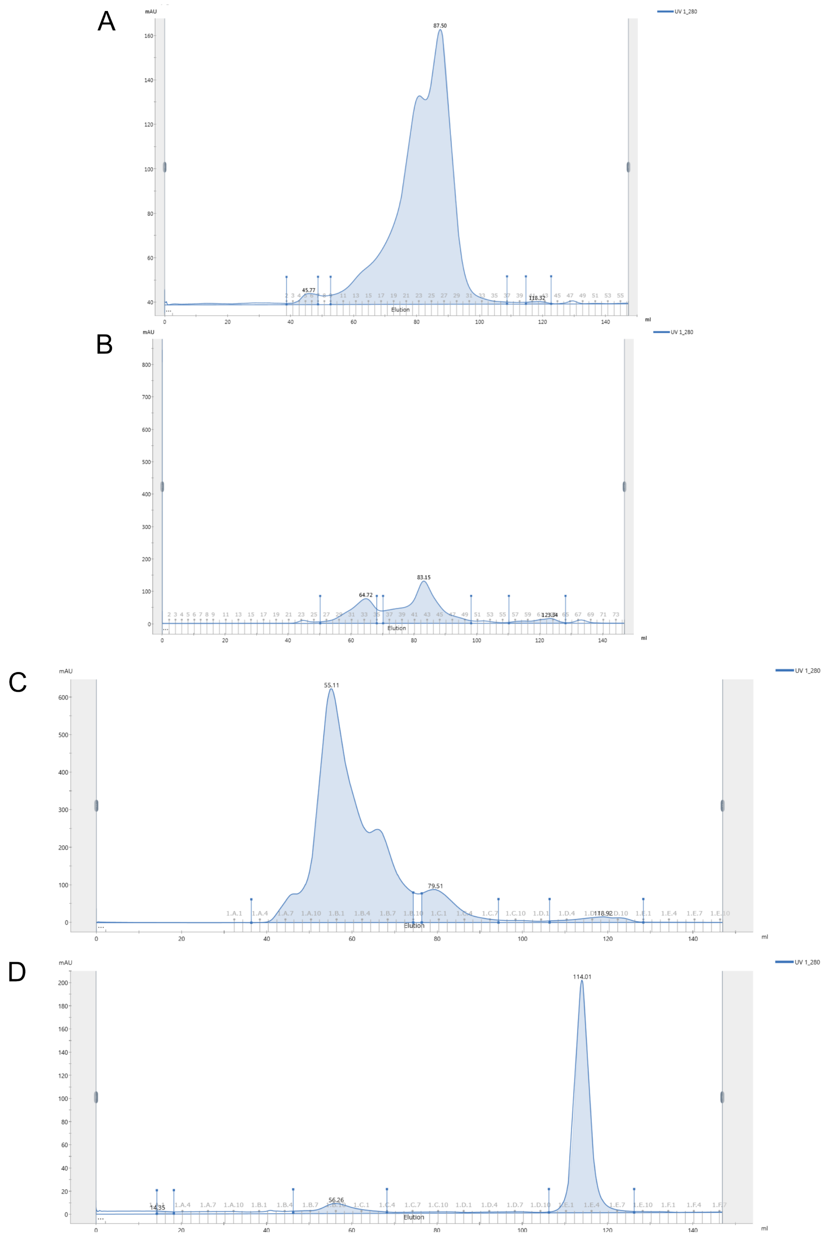

Supplementary Fig. 47 | Chromatography traces of MAS samples. All chromatograms are shown with absorbance at 280 nm. (**A**) Purification of truncated MAS KS by HiLoad 16/600 Superdex 200 pg demonstrated the equilibrium between monomeric (47.9 kDa) and dimeric (95.8 kDa) truncated MAS KS where the monomeric form is predominant. (**B**) Purification of truncated MAS KS-LD-AT by HiLoad 16/600 Superdex 200 pg demonstrated the equilibrium between monomeric (93.1 kDa) and dimeric (186.2 kDa) truncated MAS KS-LD-AT where the monomeric form is predominant. (**C**) Purification of MAS by HiLoad 16/600 Superdex 200 pg. The dimeric form (448.0 kDa) is predominant. (**D**) Purification of crosslinked MAS (with crosslinker **1**) by HiLoad 16/600 Superdex 200 pg. The dimeric form is predominant, and there was no significant amount of monomeric form was observed. Fraction B10 was subjected to cryo-EM analysis.

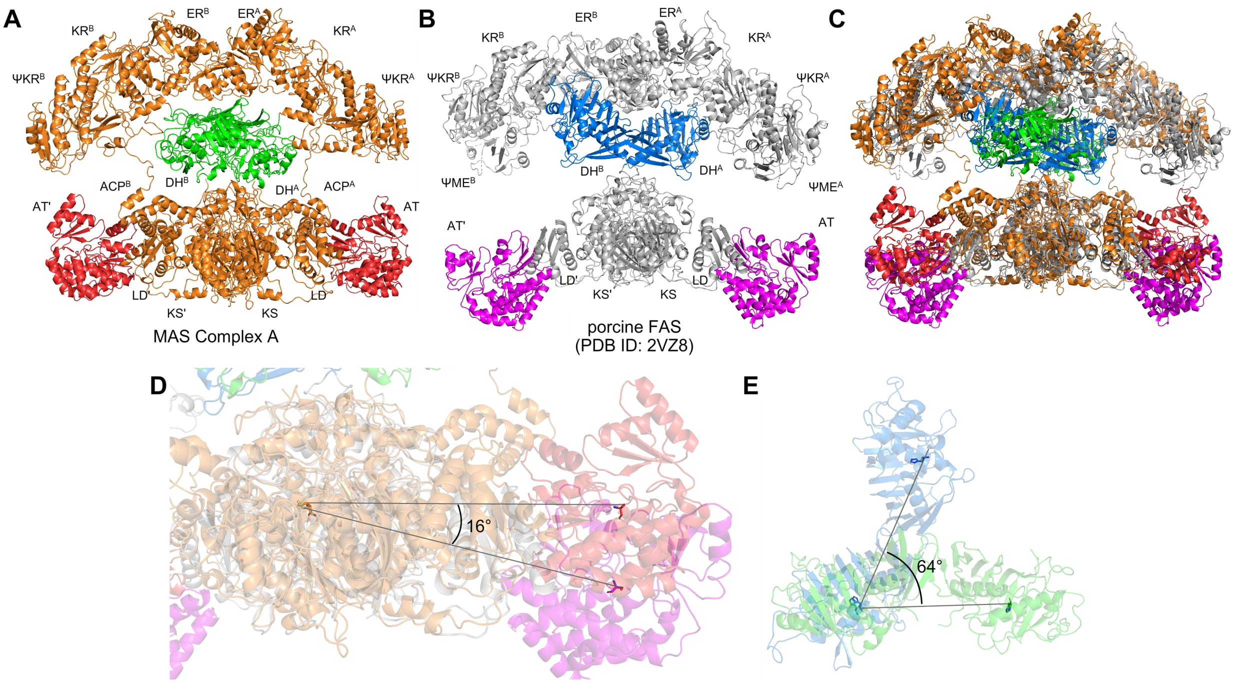

Supplementary Fig. 48 | Comparison between MAS complex A and porcine FAS. (A) (B) (C) In the alignment of porcine FAS KS dimeric core (PDBID: 2VZ8) to MAS KS dimeric core (RMSD= 1.154) showed **(D)** porcine FAS AT (magenta) bent outward with a 16° angle compared with MAS AT (red) (measured by the Cβ of the KS active residue cysteine and Cβ of the AT active residue serine). Homodimeric porcine FAS DH domain (blue) adopts a "V" shaped conformation while MAS homodimeric DH domain (green) is flat. **(E)** Alignment of porcine FAS DH to MAS DH (RMSD= 7.122) showed a 64° angle (measured by the Cβ of the DH active residue histidine and Cβ of the DH active residue histidine). Such a "V" shaped conformation of porcine FAS DH domain provides more contacts with the ER domain compared with the limited contact between MAS DH and ER as shown in **(A)** and **(B)**.

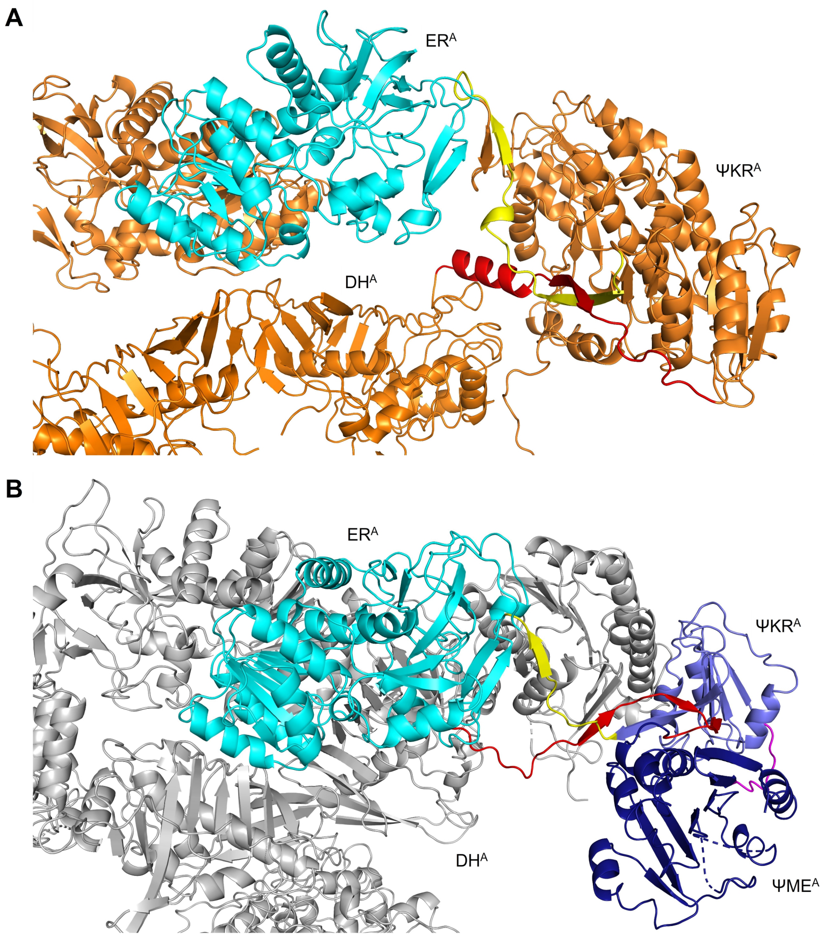

Supplementary Fig. 49 | Comparison of linkers in MAS complex A and pFAS. MAS complex A shares similarities in terms of inter-domain linkers except for the insertion of ΨME and its downstream linker. **(A)** In MAS complex A, post-DH linker (red) connects to ΨKR followed by post-ΨKR linker (yellow) connecting to ER. **(B)** In pFAS, post-DH linker (red) connects to ΨME followed by post-ΨME linker (magenta). The post-ΨME linker then connects to ΨKR followed by post-ΨKR linker (yellow) leading to ER.

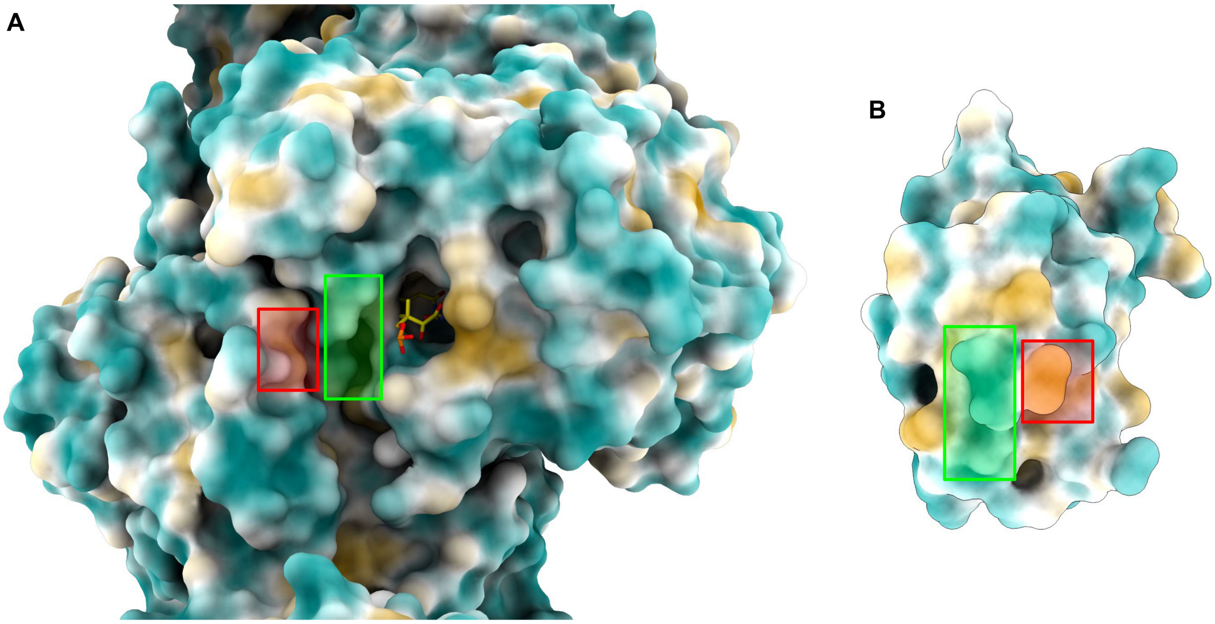

Supplementary Fig. 50 | Electrostatic and hydrophobic interactions between ACP and the KS domain. (A) (B) In complex B, besides the hydrogen bond formed between Arg2045 (ACP) and Glu79 (KS) for the interactions between ACP and the KS domain, weak hydrophobic and electrostatic interactions were also observed. Leu2061 (ACP) and Tyr146 (KS) (highlighted in red) contributed to the hydrophobic interaction while Asp2059, Gly2057 (ACP) and Ser84, Glu87 (KS) (highlighted in green) contributed to the electrostatic interactions between KS and ACP.

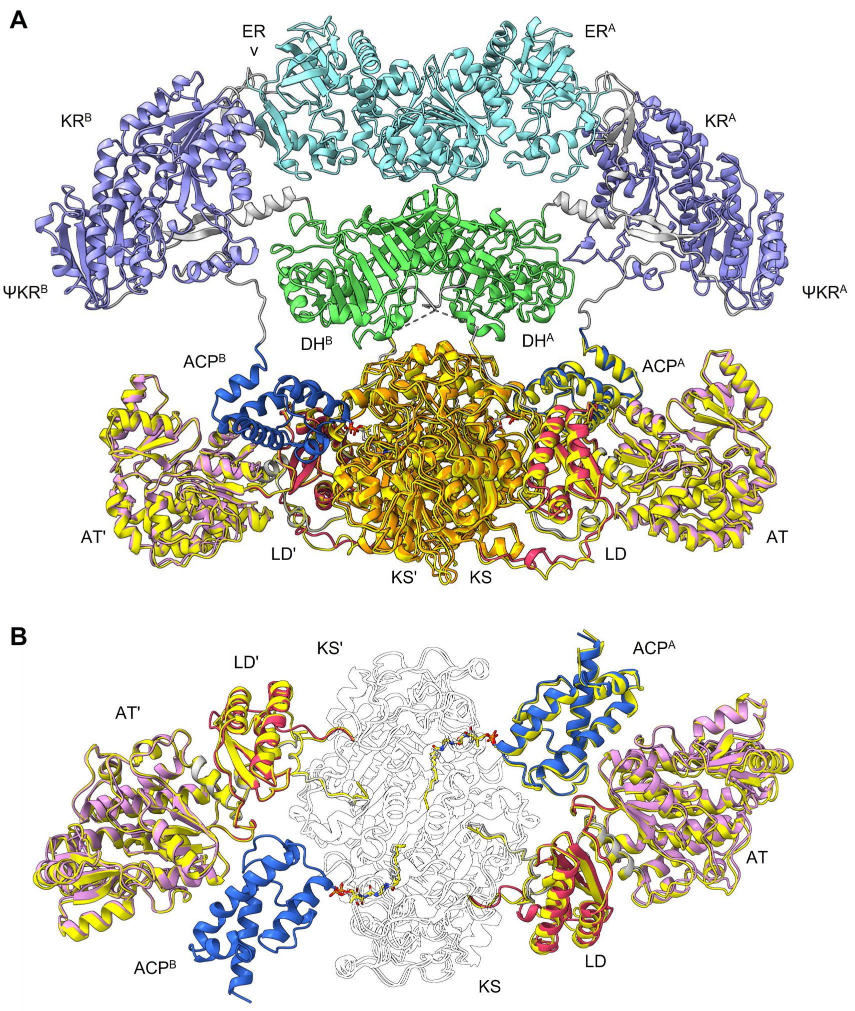

Supplementary Fig. 51 | Overall comparison between the condensation compartments in complexes A and B. (A) Alignment of complex B (yellow) to the condensation compartment of complex A (same color scheme as Fig. 2a) (RMSD= 0.705) illustrates a similar architecture of the KS-LD-AT tridomain core. **(B)** Complexes A and B adopt similar ACP docking onto KS. Crosslinker **1** in both complexes utilize the same KS binding tunnel in a similar conformation. This overlay also revealed that transacylation (substrate loading or reloading) step can be accessed by one ACP synchronously but asymmetrically or two ACPs synchronously and symmetrically.

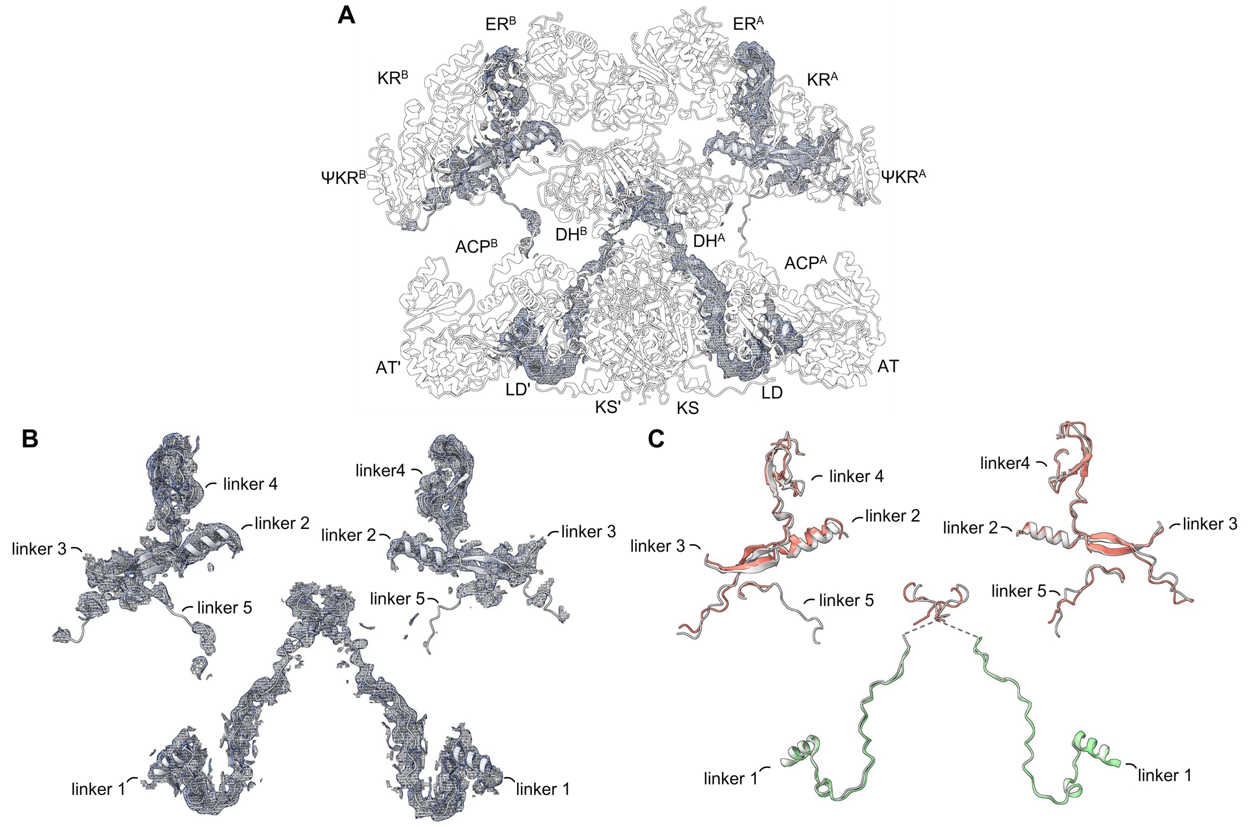

Supplementary Fig. 52 | Illustration of linkers 1-5. Panels **(A)** **(B)** visualize linkers 1-5 from complex A with density at a contour level of 0.088. **(C)** Alignment of complex B (green) to the condensation compartment of complex A (grey) (RMSD= 0.705) shows that linker 1 is in similar conformation in both complexes A and B. Alignment of complex C (salmon) to the modification compartment of complex A (grey) (RMSD= 2.901) shows similar conformation of linkers 2-4. Linker 5 in complex A (grey) attached to the ACP crosslinked at KS adopts a different conformation from the linker 5 in complex C (salmon) attached to the ACP crosslinked at DH.

Supplementary Table 1 | Cryo-EM data collection, refinement and validation statistics

	Complex A (EMDB-46504) (PDB 9D2Y)	Complex B (EMDB-46505) (PDB 9D2Z)	Complex C (EMDB-46506) (PDB 9D30)	Complex D (EMDB-46507)
**Data collection and processing**				
Magnification	130,000x	130,000x	130,000x	130,000x
Voltage (kV)	200	200	200	200
Electron exposure (e–/Å^2)	55	55	55	55
Defocus range (μm)	-0.8 to -2.0	-0.8 to -2.0	-0.8 to -2.0	-0.8 to -2.0
Pixel size (Å)	0.889	0.889	0.889	0.889
Symmetry imposed	C2, marginalized	C1	C1	C1
Initial particle images (no.)	1055614	1055614	1055614	1055614
Final particle images (no.)	35170	34615	35969	19622
Map resolution (Å)	3.87	3.22	3.74	6.15
FSC threshold	0.143	0.143	0.143	0.143
**Refinement**				
Initial model used (AlphaFold)	AF-Q02251	AF-Q02251	AF-Q02251	
Model resolution (Å)	4.4	3.6	4.1	
FSC threshold	0.5	0.5	0.5	
Map sharpening *B* factor (Å^2)	62.9	73.4	72.1	
Model composition				
Non-hydrogen atoms	31405	13794	17585	
Protein residues	4186	1855	2324	
Ligands	2	2	1	
B factors (Å^2)				
Protein	151	90.4	155	
Ligand	83.5	64.0	224	
R.m.s. deviations				
Bond lengths (Å)	0.003	0.003	0.003	
Bond angles (°)	0.646	0.678	0.603	
Validation				
MolProbity score	2.07	1.51	2.11	
Clashscore	14.20	8.62	11.62	
Poor rotamers (%)	0.12	0.28	0.72	
Ramachandran plot				
Favored (%)	93.78	97.78	90.69	
Allowed (%)	6.13	2.16	8.97	
Disallowed (%)	0.10	0.05	0.34	

Supplementary Table 2| Primers used for mutant production

MAS KS-AT gibson F primer	GTTTAACTTTAAGAAGGAGATATACATATGGAATCACGTGTCACTC
MAS KS-AT gibson F primer	GAAAATATAAATTTTCACCGGTACCTGCCCGCTGTTCTTGC
pET21 gibson F primer	GCAAGAACAGCGGGCAGGTACCGGTGAAAATTTATATTTTC
pET21 gibson F primer	GAGTGACACGTGATTCCATATGTATATCTCCTTCTTAAAGTTAAAC
MAS E79K F primer	GATGCTAAATTCTTCGGGATTAGCGAGCG
MAS E79K R primer	GAAGAATTTAGCATCGAAACCGGCGAC
MAS C177A F primer	ACCGCGGCGTCTTCGGGTCTGATGGCGGTG
MAS C177A R primer	CGAAGACGCCGCGGTGTCAAACGTCATCGCCG
MAS D274R F primer	AATCAGCGCGGCCGCACGGAGACGC
MAS D274R R primer	GCGGCCGCGCTGATTGGTGGCCGTG
MAS T279A F primer	ACGGAGGCGCTCACCATGCCGTCCGAG
MAS T279A R primer	GGTGAGCGCCTCCGTGCGGCCGTCCTG
MAS T281A F primer	ACGCTCGCCATGCCGTCCGAGGACGC
MAS T281A R primer	CGGCATGGCGAGCGTCTCCGTGCGG
MAS M417F F primer	TTCGGGTTTTCCGGGACCAACGTGCATG
MAS M417F R primer	CCCGGAAAACCCGAACGAGGACACCG
MAS M417A F primer	TTCGGGGCGTCCGGGACCAACGTGCATG
MAS M417A R primer	CCCGGACGCCCCGAACGAGGACACCG
MAS M417I F primer	TTCGGGATTTCCGGGACCAACGTGCATG
MAS M417I R primer	CCCGGAAATCCCGAACGAGGACACCG
MAS N421A F primer	GGGACCGCCGTGCATGCCATCGTGG
MAS N421A R primer	ATGCACGGCGGTCCCGGACATCCCG
MAS S623C F primer	GGACACTGCATGGGTGAGTC
MAS S623C R primer	ACCCATGCAGTGTCCGACGAC
MAS R796D F primer	GGCTACGATGTCTTCGCGGAGCTGTCG
MAS R796D R primer	GAAGACATCGTAGCCGTCCTCCATCGCAG
MAS D819R F primer	AGCCTCCGTATGTCGGTCGCCGCCCTG
MAS D819R R primer	CGACATACGGAGGCTTCGGCCCGTCTG
MAS D819K F primer	AGCCTCAAAATGTCGGTCGCCGCCCTG
MAS D819K R primer	CGACATTTTGAGGCTTCGGCCCGTCTG
MAS D819A F primer	AGCCTCGCCATGTCGGTCGCCGCCCTG
MAS D819A R primer	CGACATGGCGAGGCTTCGGCCCGTCTG
MAS H929A F primer	CGATGCGCAGGTGCATAACGTTGCC
MAS H929A R primer	CTGCGCATCGCTGAGCCAGGACAG
MAS S1187A F primer	CTAGTCGCCGAGCGGCTACTGACCCTCGG
MAS S1187A R primer	CCGCTCGGCGACTAGGCGGTCACGCTC
MAS R2032E F primer	CGGCTAGAACGTCTGCTGGTTGAGCAGG
MAS R2032E R primer	CAGACGTTCTAGCCGGCCGGCCCATTC
MAS R2045D F primer	CTGCGTGACACGATCGACGCTGACCG
MAS R2045D R primer	GATCGTGTCACGCAGGATCACACTGGCC
MAS T2046V F primer	TCGCGTGATCGACGCTGACCGCTC
MAS T2046V R primer	GATCACGCGACGCAGGATCACACTGG
MAS D2050R F primer	GACGCTCGCCGCTCATTCATCGAGTACGG
MAS D2050R R primer	TGAGCGGCGAGCGTCGATCGTGCGACG
MAS D2050K F primer	GACGCTAAACGCTCATTCATCGAGTACGG

MAS D2050K R primer	TGAGCGTTTAGCGTCGATCGTGCGACG
MAS R2090D F primer	ACCGCCGACGCTTTGGCCCAGTACTTG
MAS R2090D R primer	CAAAGCGTCGGCGGTGTTGTTTGTGGCG
